# Genetic insights into non-obstructive azoospermia: Implications for diagnosis and TESE outcomes

**DOI:** 10.1007/s10815-025-03409-5

**Published:** 2025-02-11

**Authors:** Shahrashoub Sharifi, Murat Dursun, Ayla Şahin, Serdar Turan, Ayşe Altun, Özden Özcan, Arif Kalkanlı, Kıvanç Çefle, Şükrü Öztürk, Şükrü Palanduz, Ateş Kadıoğlu

**Affiliations:** 1 Department of Internal Medicine, Division of Medical Genetics, Istanbul Medical Faculty, Istanbul, Turkey; 2 Section of Andrology, Department of Urology, İstanbul Faculty of Medicine, İstanbul University, İstanbul, Turkey; 3 Department of Obstetrics and Gynecology, İstanbul Faculty of Medicine, İstanbul University, Istanbul, Turkey; 4 Department of Urology, Medical Park Gebze Hospital, Gebze, Kocaeli, Turkey

**Keywords:** Non-obstructive azoospermia, Genetic heterogeneity, Monogenic disorders, TESE outcomes, Meiotic arrest

## Abstract

**Background:**

Non-obstructive azoospermia (NOA) is considered one of the most severe forms of male infertility. Despite the limited range of testicular phenotypes, NOA exhibits considerable genetic heterogeneity. The aim of this study was to uncover the etiopathogenesis of NOA and provide insights into the outcomes of testicular sperm extraction (TESE).

**Material method:**

To elucidate the potential causes of testicular pathogenesis, a cohort of 61 patients was analyzed. The genetic etiology was assessed using our developed gene panel, based on genes with prior functional studies conducted specifically in the context of testicular characterization.

**Results:**

Our analytical approach, built upon these findings, enabled us to explore the potential genetic causes of NOA and assess their relevance to TESE outcomes. A potential causal defect was identified in 14 genes across a total of 26 individuals (42%). Of these, three genes—MEIOB, TERB1, and USP26—had been previously described in men, while eight genes—SPO11, RBBP7, STS, RBMXL3, ZCCHC13, HUWE1, ESR1, and ABCD1—had been reported in prior studies. Additionally, three genes—*CEP85*, *NAP1L3*, and *CENPI*—had been previously described only in knockout (KO) phenotype studies, and this study represents the first identification of these genes in men.

**Conclusion:**

Interestingly, the histological findings of meiotic arrest were strongly linked to genes involved in meiosis, reinforcing the clinical diagnosis of patients in this cohort. Additionally, our study underscores the importance of refining diagnostic strategies that focus on genes associated with testicular phenotypes, which could enhance the accuracy of TESE success predictions.

**Supplementary Information:**

The online version contains supplementary material available at 10.1007/s10815-025-03409-5.

## Introduction

The prevalence of male infertility has increased by 1.67-fold over approximately 29 years, while the global population grew by 1.46-fold, highlighting a significant public health challenge [1]. Non-obstructive azoospermia (NOA), which affects approximately 1% of men worldwide, represents the most severe form of male infertility [2]. While NOA is defined by the complete absence of sperm in the ejaculate, histopathological examination reveals considerable variability in testicular morphology [3]. This variability highlights the complex nature of NOA, with underlying genetic, cellular, and environmental factors contributing to the diverse testicular phenotypes observed [4].

These diverse phenotypes reflect distinct disruptions in the complex and multifactorial processes of spermatogenesis. Transcriptomic analysis reveals that 77% of human proteins are expressed in the testis, with 9.9% of these genes exhibiting significantly higher expression in the testis compared to other tissues [5, 6]. Spermatogenesis, a highly intricate process regulated by over 2000 genes, approximately 30 to 45% of which appear to be exclusively expressed in the male germline [7]. Currently, this process is diagnosed using only two genetic tests—karyotyping for chromosomal abnormalities and Y chromosome microdeletion analysis—which together identify approximately 23% of cases [2].

Among these, sperm retrieval success rates through testicular sperm extraction (TESE) range from 40 to 60%, highlighting the limitations of current diagnostic approaches [8]. This substantial diagnostic gap leaves over 73% of NOA cases unresolved, posing significant challenges in clinical management. Patients with negative outcomes following TESE, despite unremarkable findings from routine diagnostic evaluations, often express a persistent inclination to pursue repeated TESE attempts. However, in the absence of a definitive diagnosis, clinicians lack the necessary predictive data to guide treatment decisions, often resulting in unwarranted TESE procedures. These attempts frequently attempts in unsuccessful outcomes, compounding the physical, emotional, and financial burden on patients and further emphasizing the need for advanced diagnostic methodologies [9]. Additionally, men with azoospermia exhibit a 2.9-fold increased risk of cancer compared to the general population, likely due to shared genetic pathways or mutations. Notably, this elevated risk extends beyond the affected individuals to their potential offspring and at-risk relatives [10]. These findings underscore the pivotal role of genetic diagnosis, especially in cases with prognostic implications, as it enables clinicians to provide targeted counseling and informed decision-making for individuals undergoing TESE or other therapeutic interventions. Since 2013, significant advancements in genome-based technologies, particularly whole exome sequencing (WES), have facilitated the identification of genes associated with male infertility. The identification of novel genetic variants across diverse study cohorts underscores the need for continued research in this field [11–13]. Consequently, we assert that relying solely on an identified gene panel may not be sufficient for an accurate diagnosis in patients with genetic heterogeneity. With a diagnostic yield approximately 1.5 times higher than that of targeted gene panels, WES is emerging as a more reliable first-line genetic diagnostic tool [14, 15]. In our study, we enrolled 61 men diagnosed with idiopathic NOA, all of whom had a family history characterized by a high prevalence of cancer. To identify disease-associated genes, we based our analysis on a panel of genes that we personally developed, each of which had undergone functional assays or knockout (KO) phenotype studies. By integrating results from testicular biopsies, our analysis has provided valuable insights into the genetic foundations of male infertility within this specific cohort.

## Material and methods

### The study group

This study included 61 individuals, all diagnosed with primary infertility due to idiopathic non-obstructive azoospermia (NOA) (Table 1). Individuals with monogenic disorders or genetically-driven endocrine abnormalities were excluded. All participants had a normal karyotype and were negative for Y-chromosome microdeletions. No comorbid conditions were reported. Recruitment focused on families with a high prevalence of cancer and multiple cases of infertility. Informed consent was obtained from all participants in compliance with local regulations and the ethical principles of the Declaration of Helsinki. The study protocol was approved by the institutional ethics committee, and biological samples were collected and stored according to institutional guidelines for future analyses.

**Table 1:** Clinical information of individuals

Patient	Right/left testis volume (mL)	FSH (IU/L)	Total testosterone (ng/mL)	Sperm phenotype	Testis histology	Spermatozoa can be found	Johnson score		Cryptorchidism-associated NOA
F1-III4	14/16	4.27	4.29	Azoospermia	MeA	NEG	6	NA	
F1-III5	18/16	12.29	3.92	Azoospermia	MeA	NEG	6	NA	
F2-III8	18/20	3.39	3.89	Azoospermia	MeA	NEG	6	NEG	
F2-III1	14/14	21.1	2.99	Azoospermia	MeA	NEG	6	NA	
F2-III5	NA	NA	NA	Azoospermia	NA	NEG	NA	NA	
F3-III2	14/14	24.3	3.71	Azoospermia	NA	NA	NA	NA	
F3-III6	NA	NA	NA	Azoospermia	NA	NA	NA	NA	
F4-II6	20/20	2.61	7.32	Azoospermia	MeA	NEG	6	NEG	
F4-II7	NA	NA	NA	Azoospermia	NA	NEG	NA	NA	
F5-III2	14/12	9.4	5.18	Azoospermia	SCO	NEG	2	NEG	
F5-III1	14/14	10.20	4.98	Azoospermia	PoMA	POS	7	NA	
F5-III3	NA	NA	NA	Oligospermia	Normal	Normal	Normal	NA	
F6-III1	10/10	2.67	3.18	Azoospermia	MeA	POS	6	NEG	
F6-III2	20/18	13.75	2.4	Azoospermia	HS	POS	9	NEG	
F7-III5	10/10	5.20	5.31	Azoospermia	HS	POS	9	POS	
F8-III7	8/10	78.17	6.82	Azoospermia	SCO	NEG	2	NEG	
F9-III4	5/5	29.5	0.61	Azoospermia	SCO	POS	2	NEG	
F9-III6	NA	NA	NA	Azoospermia	NA	NA	NA	NA	
F10-III3	18/20	9.95	3.76	Azoospermia	SCO	NEG	2	POS	
F10-II3	NA	NA	NA	Azoospermia	NA	NEG	NA	NEG	
F11-III2	5/5	58.9	2.59	Azoospermia	SCO	NEG	2	POS	
F11-III5	NA	NA	NA	Azoospermia	NA	NEG	NA	NA	
F12-III4	3/3	0.6	NA	Azoospermia	NA	NEG	NA	NEG	
F13-III3	12/10	19	7.52	Azoospermia	SCO	NEG	2	NEG	
F14-III2	8/8	46	NA	Azoospermia	NA	NA	NA	NEG	
F14-III3	NA	NA	NA	Azoospermia	NA	NA	NA	NA	
F15-III4	8/11	5.3	6.58	Azoospermia	SCO	NEG	2	NA	
F16-II2	NA	5.8	3.91	Azoospermia	HS	POS	9	NEG	
F17-III3	15/15	11.63	2.47	Azoospermia	NA	NEG	NA	NA	
F18-III3	12/12	6.36	5.79	Azoospermia	SCO	NEG	2	NEG	
F4-III-1	14/16	18.85	2.79	Azoospermia	SCO	NEG	2	NEG	
F20-II2	NA	NA	NA	Azoospermia	NA	NEG	NA	NA	
F21-II2	15/18	6.4	3.68	Azoospermia	HS	POS	9	NEG	
F22-III1	20/20	12.2	3.55	Azoospermia	MeA	NEG	6	NEG	
F23-III3	18/14	6.71	3.23	Azoospermia	MeA	POS	6	NEG	
F24-II3	15/15	14.2	5.03	Azoospermia	MeA	NEG	6	POS	
F25-II3	9/-	37	2.17	Azoospermia	SCO	NEG	2	POS	
F26-III3	15/15	23	3.8	Azoospermia	MeA	NEG	6	NEG	
F27-II3	15/15	15.17	1.1	Azoospermia	MeA	NEG	6	NEG	
F28-III5	24/22	2.1	4.9	Azoospermia	MeA	NEG	6	NEG	
F29-II2	-/8	44.7	4.95	Azoospermia	SCO	NEG	2	POS	
F30-II2	10/10	24.33	4.03	Azoospermia	SCO	NEG	2	NEG	
F31-III2	8/12	10.6	4.98	Azoospermia	SCO	NEG	2	NEG	
F32-III3	14/12	15.3	5.6	Azoospermia	HS	POS	9	NEG	
F33-II3	15/15	12	2.7	Azoospermia	SCO	NEG	2	NEG	
F34-II6	22/-	21.98	3.16	Azoospermia	SCO	POS	2	POS	
F35-II4	15/12	41.8	4.28	Azoospermia	SCO	NEG	2	NEG	
F36-III1	24/22	3.2	4.02	Azoospermia	MeA	NEG	6	NEG	
F37-II1	NA	NA	NA	Azoospermia	NA	NEG	NA	NA	
F38-II4	NA	NA	NA	Azoospermia	NA	NEG	NA	NA	
F39-II1	20/20	50.2	1.06	Azoospermia	SCO	POS	2	NEG	
F40-III2	14/14	7.7	4.05	Azoospermia	PoMA	POS	7	NEG	
F41-III9	13/12	13.08	4.39	Azoospermia	NA	NEG	NA	NEG	
F42-II3	NA	17.08	1.51	Azoospermia	NA	NEG	NA	NEG	
F43	16/14	1.94	6.71	Azoospermia	HS	POS	9	NEG	
F44	14/10	39.8	2.69	Azoospermia	SCO	NEG	1	NEG	
F45	12/10	44.79	3.05	Azoospermia	SCO	NEG	2	NEG	
F46	18/14	6.85	4.61	Azoospermia	SCO	NEG	2	NEG	
F47	18/16	13.95	7.77	Azoospermia	SCO	NEG	2	NEG	
F48	22/22	3.67	6.8	Azoospermia	MeA	NEG	6	NEG	
F49	10/10	26.3	4.57	Azoospermia	SCO	NEG	2	NEG	

### Tissue collection and histological assessment

Testicular tissue samples were obtained during routine therapeutic testicular sperm extraction (TESE) procedures from men diagnosed with non-obstructive azoospermia who consented to undergo micro-TESE (mTESE) as part of the study. The procedure was performed using a microscope with 15 × magnification to enhance visualization and ensure precise tissue extraction from seminiferous tubules. The excised tissue was immediately fixed in 10% Bouin’s solution overnight to preserve cellular and tissue structure. Following fixation, the tissue samples were embedded in paraffin after undergoing dehydration through a graded ethanol series and clearing in xylene. Sections of 5-μm thickness were cut from the paraffin blocks. For histological analysis, the sections were deparaffinized by immersion in xylene for 1 h and rehydrated through graded ethanol solutions. After rehydration, the slides were stained with hematoxylin and eosin (H&E). The stained tissue sections were examined under a light microscope at magnifications of × 200 and × 400. Morphological evaluations focused on the assessment of seminiferous tubules, with a minimum of 10 representative fields selected per section. At least 50 seminiferous tubules were analyzed per section according to the Johnson score at × 15 magnification to evaluate spermatogenesis. This scoring method was used to assess the quality of spermatogenesis based on the appearance of germ cells and their maturation within the tubules.

### Development of a gene panel for predictive insights

To develop this gene panel, we conducted an extensive 25-year literature review, focusing on the molecular mechanisms of spermatogenesis. Genes associated with “meiosis,” “male infertility,” “azoospermia,” and “fertilization failure” were prioritized based on their clinical relevance and diagnostic value. Additionally, some genes associated with both azoospermia and other spermatic phenotypes were included, acknowledging variable expressivity and incomplete penetrance. To enhance the panel’s predictive accuracy, we integrated functional data from model organisms, specifically incorporating knockout phenotype studies in mice.

### Whole-exome sequencing and variant filtering

Genomic DNA was extracted from peripheral blood using the QIAamp DNA Mini Kit (QIAGEN), and sequencing was performed using the Twist Exome 2.0 kit, targeting coding regions and splice sites. Sequencing was conducted on the Illumina NovaSeq 6000 platform. Alignment, variant calling, classification, and analysis were performed using the SEQ Platform (Genomize Inc.). FASTQ files were uploaded to the SEQ Platform and processed by aligning the reads to the human reference genome GRCh37 using Burrows-Wheeler Aligner [16]. Aligned reads were used for variant calling using FreeBayes [17], PCR deduplication, and in-del realignment using proprietary algorithms by Genomize. Obtained variants were annotated using VEP v102 [18]. ACMG pathogenicity classification was performed using Genomize’s proprietary algorithm based on the guidelines published by Richards et al. (2015) [19]. Variants were cross-referenced with clinical databases such as ClinVar (2024–03–07) and dbSNP v154 (2020–05–14), alongside population databases like gnomAD, the 1000 Genomes Project, and ExAC, to assess variant frequency. Variants were filtered based on stringent criteria, including read depth (≥ 20 ×), quality score (Phred ≥ 30), and minor allele frequency (MAF < 1%), prioritizing high-impact mutations such as stop-gain, nonsense, and frameshift variants. CNV analyses were performed using the SEQ Platform (Genomize Inc.). Reads aligned to the human reference genome GRCh37 were used for CNV detection by the GATK gCNV tool v4.1.8.1 [20] with optimized parameters.

### Sanger verification of candidate variants

To validate the pathogenicity of candidate variants and clarify inheritance patterns, analyses were performed using the ABI 3500 Sequencing System. Sequence data were interpreted with SeqScape software (Applied Biosystems). To assess variant specificity, a control cohort of 300 individuals with normal sperm parameters and no history of NOA or male infertility was also screened.

## Results

### Identification of candidate genes

**Fig. 1:**
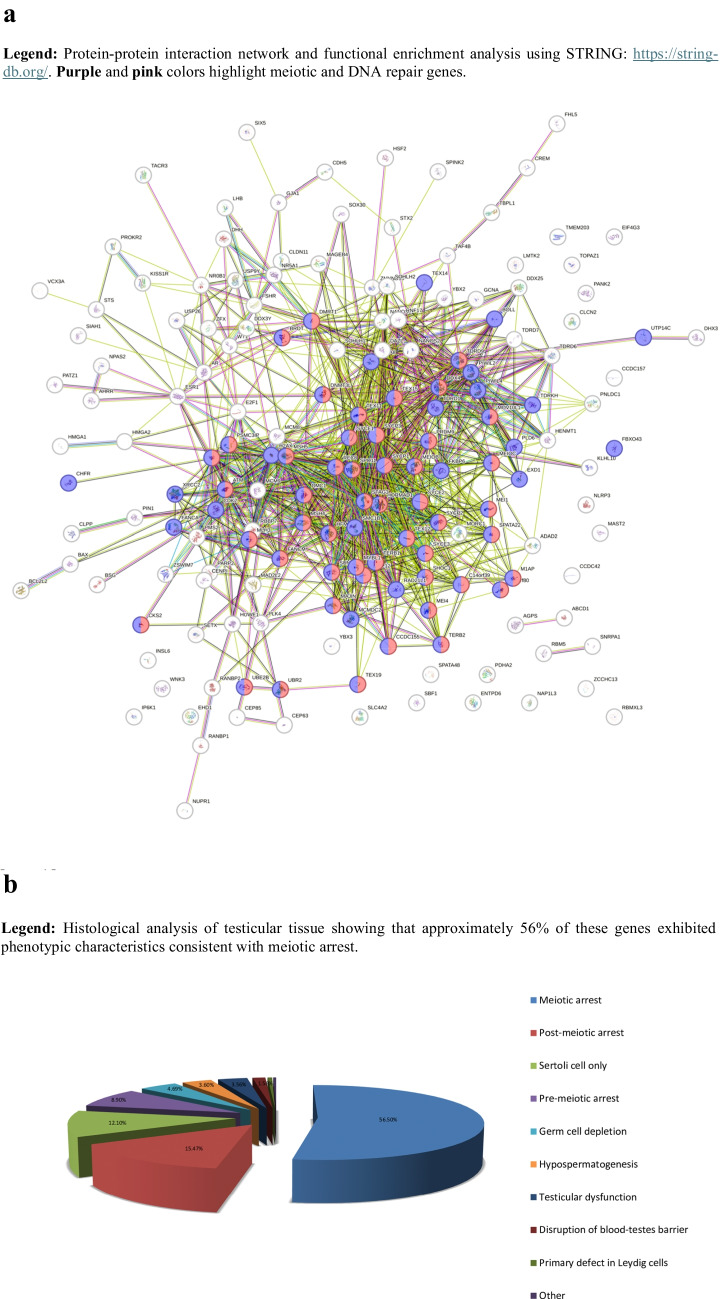
**A** Predicted protein–protein interaction analysis of proteins encoded by the 175 candidate genes in the NOA panel using STRING. *Legend: protein–protein interaction network and functional enrichment analysis of the 175 candidate genes encoded in the NOA panel, highlighting meiotic and DNA repair genes within the figure.* **b** Literature-based overview of NOA testicular phenotypes

The initial search yielded a substantial volume of manuscripts. To refine the scope, we applied a systematic exclusion process, removing studies on chromosomal abnormalities, Y-chromosome microdeletions, CNVs involving the X and Y chromosomes, monogenic disorders, multifactorial diseases, epigenetic factors, and inflammatory syndromes. This filtering process narrowed the dataset to approximately 1200 manuscripts, which were subjected to detailed analysis. In this step, we extracted key data on candidate genes, their roles in relevant biological pathways, the animal models used in the studies, and any functional assays performed. Additional information was collected on histological findings, phenotypic comparisons between human and animal models, inheritance patterns, and allele frequencies in different human populations. We also evaluated whether these gene abnormalities were familial or novel. Among the identified candidate genes, we focused on 175 genes most strongly associated with the molecular mechanisms underlying NOA (Supplementary Table 1). A Biological Process Gene Ontology (GO) analysis of these genes revealed that more than 60% were involved in meiotic processes, such as meiotic cell cycle regulation and DNA repair (Fig. 1a) [21, 22]. Protein–protein interaction analyses further support that these genes cluster within the same biological pathway (https://string-db.org). Moreover, histological analysis of testicular tissue further supported these findings, showing that approximately 56% of these genes exhibited phenotypic characteristics consistent with meiotic arrest (Fig. 1b).

### Exome sequencing analysis, prioritized variants

Based on our gene panel, we prioritized and classified variants using over 20 in silico pathogenicity prediction tools (Supplementary 2). Variants with high pathogenicity scores were further analyzed to determine their allele frequencies across multiple population databases (Supplementary 2). High-frequency variants were excluded at this stage. Notably, two variants that were absent in gnomAD and had high pathogenicity scores were initially identified. However, family segregation analysis revealed their presence in healthy individuals, indicating that these variants were benign, and they were subsequently excluded. These variants were submitted to ClinVar as benign. In contrast, variants with lower prediction scores but absent or exceptionally rare in population databases were further evaluated. These variants were analyzed in the patient cohort, among affected family members, and within the control group to determine their potential pathogenic significance. In the second phase, for individuals in whom no potential variants were identified, we expanded the analysis beyond our developed panel. We focused on testis-specific genes and their associated molecular pathways. As a result, we identified two strong candidate genes, which were planned for functional assays (Table 2). Segregation analysis was conducted for all available participants in the study to assess pathogenicity confidence and determine whether the variants were inherited in trans, maternally transmitted, or represented de novo mutations.

**Table 2:** List of identified candidate gene variants

Gene	Types of mutations	Genotype-Tissue Expression	OMIM	Locus	Inheritance	HGVSc	HGVSp	GnomAD	Segregation in related patients
TERB1	Frameshift	Testis	617,332	chr16:66,809,045 CA- > C	AR	ENST00000433154.1:c.1086del	ENSP00000463762.1:p.Phe362LeufsTer15	0	Yes
MEIOB	Stop Gained	Testis	617,670	chr16:1,903,229 G- > A	AR	ENST00000412554.2:c.673C > T	ENSP00000390778.2:p.Arg225Ter	0.000006183	Yes
USP26	Stop Gained	Testis	300,309	chrX:132,162,218 G- > A	XLR	ENST00000511190.1:c.31C > T	ENSP00000423390.1:p.Gln11Ter	0	Yes
SPO11	Stop Gained	Testis	605,114	chr20:55,913,366 T- > G	AR	ENST00000371263.3:c.779 T > G	ENSP00000360310.3:p.Leu260Ter	0	Yes
CEP85	Inframe Insertion		618,898	chr1:26,570,713 A- > ATCTCGGAGCCCT	AR	ENST00000252992.4:c.114_125dup	ENSP00000252992.4:p.Ser39_Phe42dup	0	
STS	Missense		300,747	chrX:7,268,102 G- > A	XLR	ENST00000217961.4:c.1552G > A	ENSP00000217961.4:p.Ala518Thr	0.0005172	Yes
CENPI	Missense		300,065	chrX:100,383,754 A- > G	XLR	ENST00000372927.1:c.1124A > G	ENSP00000362018.1:p.Tyr375Cys	0.0001714	Yes
ZCCHC13	Missense	Testis	301,125	chr9:841,866 C- > T	XLR	ENST00000339534.2:c.263G > A	ENSP00000345633.2:p.Arg88His	0.00006579	
RBBP7	Missense		300,825	chrX:16,864,045 T- > G	XLR	ENST00000380084.4:c.1247A > C	ENSP00000369424.4:p.His416Pro	0	Yes
HUWE1	Missense		300,697	chrX:53,674,465 G- > A	XLR	ENST00000342160.3:c.197C > T	ENSP00000340648.3:p.Ala66Val	0	Yes
RBMXL3	Missense	Testis	300,199	chrX:114,425,479 G- > T	XLR	ENST00000424776.3:c.1475G > T	ENSP00000417451.2:p.Ser492Ile	0	Yes
ESR1	Missense		133,430	chr6:152,265,380 G- > T	AR	ENST00000440973.1:c.833G > T	ENSP00000405330.1:p.Gly278Val	0.00009797	Yes
NAP1L3	Missense		300,117	chrX:92,927,339 A- > G	XLR	ENST00000373079.3:c.965 T > C	ENSP00000362171.3:p.Val322Ala	0	
ABCD1	Missense		300,371	chrX:153,001,803 C- > T	XLR	ENST00000218104.3:c.1229C > T	ENSP00000218104.3:p.Thr410Met	0.00002951	

### Identified gene variants

Among the analyzed individuals, CNV analyses were found to be normal for the entire study group. Variants potentially associated with the disease were identified in 26 out of 61 patients. Of these, 33.3% exhibited X-linked recessive (XLR) inheritance, while 66.7% followed an autosomal recessive (AR) inheritance pattern. Notably, 58% of these variants are absent in gnomAD (Table 2) (Supplementary 3).

Loss-of-function (LoF) variants in genes—MEIOB (MIM: 617,670), TERB1 (MIM: 617,332), USP26 (MIM: 300,309), and SPO11 (MIM: 605,114)—previously associated with NOA, were identified in 10 individuals from four families (Table 2).

Additionally, we identified 15 individuals with missense variants in nine genes involved in critical processes of spermatogenesis (Table 2). These genes, including RBBP7 (MIM: 300,825), HUWE1 (MIM: 300,697), RBMXL3 (MIM: 300,199), CENPI (MIM: 300,065), STS (MIM: 300,747), and ESR1 (MIM: 133,430), were identified in 10 individuals from five distinct families.

Variants in RBBP7, HUWE1, and RBMXL3 were absent from gnomAD, with pathogenicity scores of 21, 6, and 4 out of 22, respectively. Similarly, variants in CENPI, STS, and ESR1 were very rare in gnomAD. Predicted pathogenicity scores were 16, 3, and 5 out of 22 prediction tools, respectively.

In contrast to the other variants, ABCD1 (MIM: 300,371), NAP1L3 (MIM: 300,117), and ZCCHC13 (MIM: 301,125) were exclusively identified in single individuals from three distinct families. The NAP1L3 variant, which was absent from population databases, generated a score of 9 out of 22 prediction tools. Similarly, variants in ABCD1 and ZCCHC13 were extremely rare in gnomAD, with scores of 20 and 3 out of 22, respectively.

Furthermore, one individual carried a homozygous in-frame insertion in the CEP85 (MIM: 618,898) gene. This variant, which was exclusively observed in this individual, was absent in population databases and was predicted to be pathogenic according to both ACMG guidelines and VarSome database [23].

It is important to emphasize that functional studies provided compelling evidence for the role of these genes in NOA-related male sterility. A comprehensive analysis of individuals, including both affected and unaffected family members, as well as 300 control individuals, led to a definitive genetic diagnosis for 10 individuals. For 16 individuals, a probable diagnosis was established based on variants not previously reported.

### Histological phenotypes and testicular sperm extraction (TESE) outcomes

**Fig. 2:**
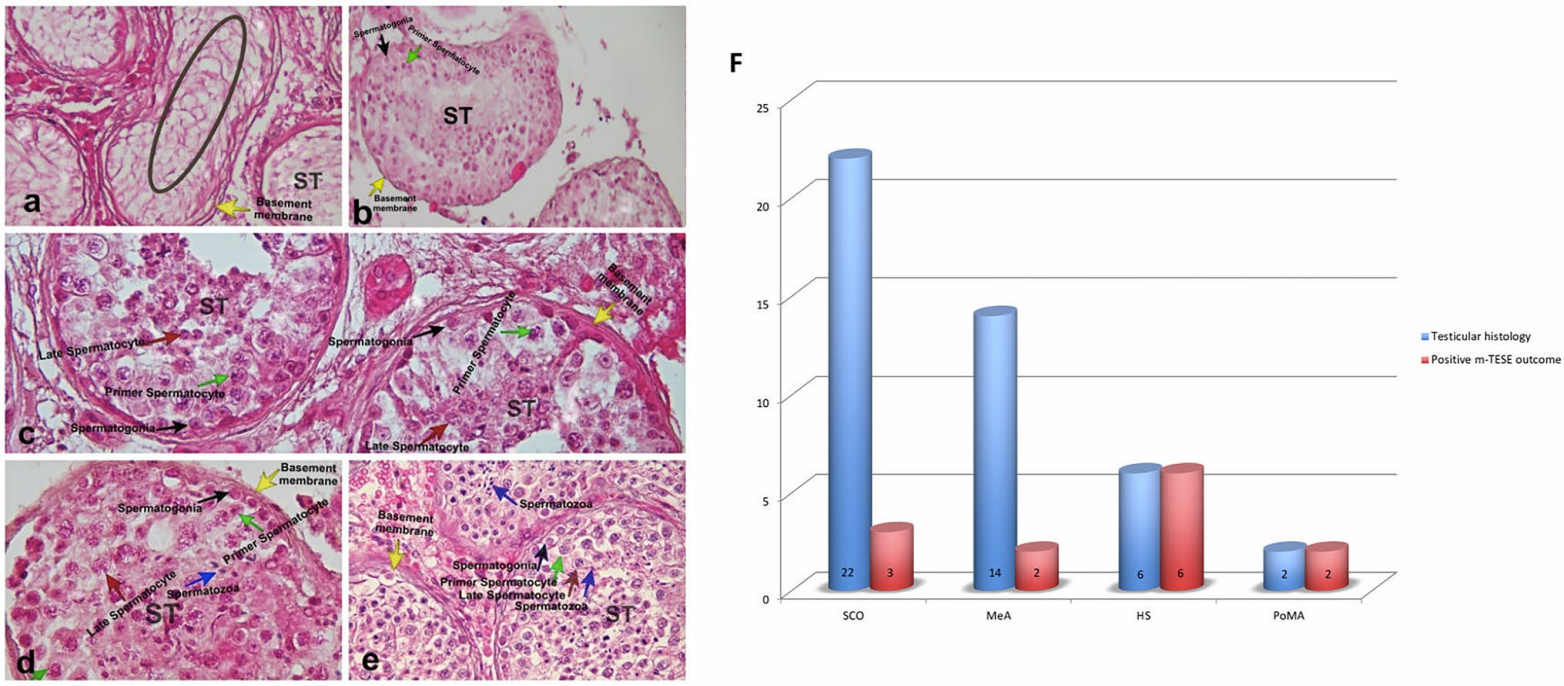
**a**, **b**, **c**, **d**, and **e** Histological micrographs of testis biopsy samples. **f** Correlation between testicular histology and successful TESE outcomes. Legend: **a** Sertoli cell-only syndrome (SCOS): The tubules contain only Sertoli cells, with no germ cells present. **b** Maturation arrest at the primary spermatocyte stage (MA) is characterized by tubule structures where spermatogonial cells are positioned near the base of the tubules. **c** Post-meiotic arrest (post-MA): The image shows seminiferous tubule structures where no sperm are observed, but numerous spermatids are present. **d** Hypospermatogenesis (HS): Tubule structures where no complete tubule formation is observed, but a few sperm cells are present. **e** Normal spermatogenesis (NS): The image shows seminiferous tubule structures where cells in various stages of spermatogenesis are observed. Yellow arrow: basement membrane, black arrow: spermatogonia, green arrow: primary spermatocyte, red arrow: late spermatocyte, blue arrow: spermatozoon, circle: empty seminiferous tubule lumen, and ST, seminiferous tubule

In this cohort study, testicular histology and sperm retrieval outcomes were assessed in 45 of the 61 NOA patients (Table 3) (Fig. 2a, b, c, d, and e). Among the 22 patients with SCOS, sperm retrieval was successful in three cases, yielding a success rate of 13.63%, with an average Johnson score of 2. In the MeA group, which included 14 patients, sperm retrieval was successful in two cases, resulting in a success rate of 14.28%, with moderate Johnson scores averaging 6. Notably, two patients diagnosed with PoMA also achieved successful sperm retrieval, with Johnson scores averaging 7, although this diagnosis was less common in the study. In contrast, all six patients with HS achieved successful sperm retrieval, resulting in a 100% success rate, with consistently high Johnson scores averaging 9 (Fig. 2f).

**Table 3:** Clinical information of affected individuals

Patient	Gene	Function	Right/left testis volume (mL)	FSH (IU/L)	Total testosterone (ng/ml)	Sperm phenotype	Testis histology	Spermatozoa can be found	Johnson score	Cryptorchidism-associated NOA
F1-III4	SPO11	Initiates meiosis by introducing double-strand breaks in DNA	14/16	4.27	4.29	Azoospermia	MeA	NEG	6	NA
F1-III5	SPO11	Initiates meiosis by introducing double-strand breaks in DNA	18/16	12.29	3.92	Azoospermia	MeA	NEG	6	NA
F2-III8	MEIOB	Facilitates homologous recombination and DNA repair during meiosis I	18/20	3.39	3.89	Azoospermia	MeA	NEG	6	NEG
F2-III1	MEIOB	Facilitates homologous recombination and DNA repair during meiosis I	14/14	21.1	2.99	Azoospermia	MeA	NEG	6	NA
F2-III5	MEIOB	Facilitates homologous recombination and DNA repair during meiosis I	NA	NA	NA	Azoospermia	NA	NEG	NA	NA
F3-III2	STS	Hydrolyzes steroid sulfates, influencing steroid hormone metabolism	14/14	24.3	3.71	Azoospermia	NA	NA	NA	NA
F3-III6	STS	Hydrolyzes steroid sulfates, influencing steroid hormone metabolism	NA	NA	NA	Azoospermia	NA	NA	NA	NA
F4-II6	TERB1	Synaptonemal complex formation for homologous chromosome pairing in meiosis	20/20	2.61	7.32	Azoospermia	MeA	NEG	6	NEG
F4-II7	TERB1	Synaptonemal complex formation for homologous chromosome pairing in meiosis	NA	NA	NA	Azoospermia	NA	NEG	NA	NA
F5-III2	USP26	Regulate protein turnover and aid germ cell movement across the blood-testis barrier before meiosis	14/12	9.4	5.18	Azoospermia	SCO	NEG	2	NEG
F5-III1	USP26	Regulate protein turnover and aid germ cell movement across the blood-testis barrier before meiosis	14/14	10.20	4.98	Azoospermia	PoMA	POS	7	NA
F5-III3	USP26	Regulate protein turnover and aid germ cell movement across the blood-testis barrier before meiosis	NA	NA	NA	Oligospermia	Normal	Normal	Normal	NA
F6-III1	CENPI	Formation of functional centromeres and in mitosis	10/10	2.67	3.18	Azoospermia	MeA	POS	6	NEG
F6-III2	CENPI	Formation of functional centromeres and in mitosis	20/18	13, 75	2, 4	Azoospermia	HS	POS	9	NEG
F7-III5	NAP1L3	Histone chaperone that aids nucleosome assembly and chromatin remodeling	10/10	5.20	5.31	Azoospermia	HS	POS	9	POS
F8-III7	ZCCHC13	Activates AKT/MAPK/c-MYC pathway in male germ cells, cell cycle regulation and proliferation	8/10	78.17	6.82	Azoospermia	SCO	NEG	2	NEG
F9-III4	RBBP7	Chromatin remodeling factor, regulation of cell proliferation and differentiation	5/5	29.5	0.61	Azoospermia	SCO	POS	2	NEG
F9-III6	RBBP7	Chromatin remodeling factor, regulation of cell proliferation and differentiation	NA	NA	NA	Azoospermia	NA	NA	NA	NA
F10-III3	HUWE1	Regulator of DNA damage response,cellular stress responses, transcription, autophagy, apoptosis, and metabolism, a critical regulator of spermatogonial differentiation	18/20	9.95	3.76	Azoospermia	SCO	NEG	2	POS
F10-II3	HUWE1	Regulator of DNA damage response, cellular stress responses, transcription, autophagy, apoptosis, and metabolism, a critical regulator of spermatogonial differentiation	NA	NA	NA	Azoospermia	NA	NEG	NA	NEG
F11-III2	RBMXL3	RNA-binding protein involved in the regulation of alternative splicing	5/5	58.9	2.59	Azoospermia	SCO	NEG	2	POS
F11-III5	RBMXL3	RNA-binding protein involved in the regulation of alternative splicing	NA	NA	NA	Azoospermia	NA	NEG	NA	NA
F12-III4	CEP85	Regulates centrosome cohesion and spindle orientation in cell division, promote mitotic spindle assembly	3/3	0.6	NA	Azoospermia	NA	NEG	NA	NEG
F13-III3	ABCD1	Peroxisomal import of fatty acids and/or fatty acyl-CoAs in the organelle	12/10	19	7.52	Azoospermia	SCO	NEG	2	NEG
F14-III2	ESR1	Nuclear receptor superfamily of transcription factors, primarily regulated by binding of estrogen/estradiol (E2)	8/8	46	NA	Azoospermia	NA	NA	NA	NEG
F14-III3	ESR1	Nuclear receptor superfamily of transcription factors, primarily regulated by binding of estrogen/estradiol (E2)	NA	NA	NA	Azoospermia	NA	NA	NA	NA

### Phenotype-related mutations

Mutations in several key genes were identified in 26 patients. In the meiosis pathway, mutations in SPO11, MEIOB, and TERB1 were found in 7 patients, with a high prevalence of 26.9%, all of which resulted in meiotic arrest (MeA) and negative testicular sperm extraction (TESE) (Table 3).

In the chromatin remodeling and gene regulation pathway, mutations in NAP1L3F, RBBP7, and RBMXL3 were found in 5 patients. The NAP1L3F mutation was associated with hypospermatogenesis and a positive TESE result. Mutations in RBBP7 and RBMXL3 led to Sertoli cell-only syndrome (SCOS). While RBBP7 was associated with a positive TESE result, RBMXL3 was associated with a negative TESE result in both cases.

In the hormonal and steroid metabolism pathway, mutations in ESR1 and STS were found in 4 patients, all of whom exhibited NOA. TESE was not performed for this group.

In the spermatogonial differentiation and blood-testis barrier pathway, mutations in USP26 affected 3 patients from the same family, leading to diverse outcomes. These included Sertoli cell-only syndrome (SCOS) with negative TESE, post-meiotic arrest (PoMA) with positive TESE, and oligospermia.

In the centromere function and cell division pathway, mutations in CENPI were identified in 2 patients from the same family, one presenting with meiotic arrest and the other with hypospermatogenesis. Both patients tested positive for TESE.

In the DNA damage and cellular stress response pathway, mutations in HUWE1 were observed in 2 patients from the same family, one of whom exhibited Sertoli cell-only syndrome (SCOS). For the other patient, the testicular phenotype was not available. Both patients had negative outcomes for TESE.

Mutations in CEP85 in the centrosome cohesion and spindle assembly pathway were identified in one patient, resulting in a negative TESE. Similarly, mutations in ABCD1 in the fatty acid metabolism pathway were present in one patient, leading to Sertoli cell-only syndrome (SCOS) and a negative TESE outcome. Notably, despite positive sperm retrieval outcomes in some cases, none of the patients achieved a clinical pregnancy.

## Discussion

Non-obstructive azoospermia (NOA) is one of the most intricate and poorly understood causes of male infertility [24]. The pathophysiological mechanisms underlying NOA exhibit considerable heterogeneity, with significant interindividual variability [25]. Notably, the genetic basis of NOA extends beyond chromosomal abnormalities and Y-chromosome microdeletions, which collectively account for fewer than 23% of diagnosed cases [2].

In recent years, whole-exome sequencing (WES) analyses have advanced the field, enabling the identification of numerous candidate genes implicated in the pathogenesis of NOA [26, 27]. The identification of new candidate genes in each study underscores the need for continued research in this field [12, 28–33]. Focusing exclusively on a single set of detected genes may lead to a loss in diagnostic yield, potentially reducing diagnoses by 1.5 times [14, 15].

The human genome contains over 6 billion bases, making it too complex for experiments to focus on each base individually. Information from diverse populations can identify frequent variants in patients and facilitate the identification of candidate genes. On the other hand, single-point variants may exhibit phenotypes distinct from those observed in knockout mouse models. Therefore, genome sequencing methods remain critically important.

For men with NOA, molecular diagnosis via exome sequencing is not yet routinely recommended, primarily due to the lack of specific treatment algorithms tailored to identified genetic variants. However, 56% of the genes in our 175-gene panel are implicated in meiotic processes. Robust evidence supports that defects in these genes play a critical role in the pathogenesis of meiotic arrest, underscoring their importance in the genetic etiology of NOA. Furthermore, numerous studies have demonstrated strong genotype–phenotype correlations, particularly highlighting that mutations in meiotic genes are strongly linked to negative TESE outcomes [12, 26, 34].

These findings emphasize the need to create clear diagnostic and treatment guidelines to incorporate WES into regular clinical practice. In our study, based on this knowledge, we evaluated the potential gene defects associated with NOA and their relationship to the testicular phenotype. Histological analysis revealed four distinct types of testicular defects, with Sertoli cell-only syndrome (SCOS) being the most prevalent, affecting 22 patients, followed by meiotic arrest in 14 cases. A significant correlation was observed between testicular histology and sperm extraction outcomes (TESE). Notably, all patients with hypospermatogenesis had a successful TESE result (100%). Additionally, two patients with post-maturation arrest exhibited successful sperm retrieval. However, due to the limited sample size, it is challenging to draw definitive conclusions regarding the success rate in this specific subgroup. Notably, the success rates were considerably lower in patients with Sertoli cell-only syndrome (13.63%) and meiotic arrest (14.28%).

These findings are in approximate concordance with those reported in similar clinical investigations. In patients with Sertoli cell-only syndrome (SCOS), the success rate of sperm retrieval through TESE ranges from approximately 6.3 to 41%, depending on the technique employed [35, 36]. For cases of meiotic arrest, the success rate for sperm retrieval varies between 27 and 86%, with outcomes influenced by whether the arrest occurs early or late in the process [35, 37].

Subsequently, we performed whole-exome sequencing analyses on 61 individuals with NOA and four types of testicular histology. Our analysis identified potential genetic defects in 26 patients (42%), with 84% of these cases demonstrating a familial inheritance pattern. This high familial association significantly enhanced the diagnostic yield in this heterogeneous cohort. Compared to conventional genetic testing, our study’s yield of 42% represents an increase of 82.6%. Similarly, a study by Kherraf ZE et al. reported a diagnostic rate of 23% (22 out of 96 individuals) using exome sequencing, further highlighting the potential of WES to nearly double the diagnostic efficiency for NOA [26].

Despite the presence of four testicular phenotypes, genetic defects were identified across several different pathways. Genes related to meiosis, including TERB1, MEIOB, and SPO11, showed the highest prevalence, with 26% of affected individuals. From a genotype–phenotype perspective, this group exhibited significant homogeneity. Similar to findings from other studies, loss-of-function mutations in these genes were associated with meiotic arrest and negative TESE outcomes [26, 38–50]. The genetic diagnostic efficiency was particularly high in patients with meiotic arrest due to the well-established genetic markers. In these cases, we can confidently diagnose a negative outcome for TESE.

Other genes associated with different biological processes, including USP26, RBBP7, ZCCHC13, RBMXL3, NAP1L3, CEP85, CENPI, STS, ESR1, HUWE1, and ABCD1 showed weak genotype–phenotype correlations.

Although mutations in the RBBP7 gene in our cohort are associated with Sertoli cell-only syndrome (SCOS) and azoospermia, previous studies have shown that mutations in different variants of this gene are linked to maturation arrest. In terms of spermatogenic phenotype, other mutations have been implicated in oligospermia and cryptozoospermia, in addition to azoospermia [32, 51, 52].

Similarly, in individuals with the RBMXL3 mutation, our findings align with previous studies, demonstrating an association with SCOS and azoospermia. However, other studies have also identified a relationship between mutations in this gene and asthenozoospermia [28, 29, 33].

The HUWE1 gene, in individuals with Sertoli cell-only syndrome (SCOS), is consistently associated with azoospermia, as reported in other studies. However, we were unable to find detailed testicular phenotype information related to this gene in the literature [30–32].

Similarly, the ZCCHC13 gene in our patient is associated with SCOS, as documented in the literature. Regarding the spermatogenic phenotype, various mutations in this gene have been linked to oligospermia and oligoasthenoteratozoospermia [28, 29, 33].

In our study, there was no information on the testicular phenotype related to STS and ESR1. However, there is a correlation between the STS gene and the spermatogenic phenotype reported in the literature [53]. In contrast, studies have linked ESR1 mutations with both azoospermia and severe oligospermia due to different gene defects [42, 54].

The ABCD1 gene in our patient, associated with Sertoli cell-only syndrome (SCOS), aligns with studies indicating severe impairment of spermatogenesis [55, 56].

Mutations in NAP1L3 identified in our patient with hypospermatogenesis have not been previously reported in men. However, animal studies have highlighted the crucial role of this gene in type A spermatogonia and Sertoli cells [57, 58]. This represents the first report of NAP1L3 mutations in men.

CEP85 is another gene that, to our knowledge, is being reported for the first time in men. This gene is essential for regulating centrosome cohesion and spindle orientation during cell division, thereby facilitating mitotic spindle assembly. In our study, no testicular phenotype associated with this gene was observed. However, co-phenotype studies have linked mutations in CEP85 to spermatogenic arrest and sterility [59, 60].

The gene defects observed in our cohort exhibit phenotypic differences when compared to those reported in the literature, which may be attributed to variations in specific mutations within the same genes or mutations at different locations within the gene. A noteworthy finding was the substantial phenotypic variability among family members who carried the same genetic variant. USP26, a gene involved in protein regulation and spermatogonial differentiation, demonstrated considerable phenotypic diversity. One patient with a USP26 mutation had Sertoli cell-only syndrome (SCOS) and no sperm, while another exhibited normal spermatogenesis with successful sperm retrieval, and a third showed post-meiotic arrest (PoMA) with sperm retrieved via TESE. Previous studies have also associated USP26 mutations with maturation arrest as a testicular phenotype, though other sperm-related issues, such as azoospermia, oligozoospermia, and asthenoteratozoospermia, have also been documented [61–67]. Similarly, mutations in CENPI, a gene essential for centromeric cohesion and spindle assembly, exhibited notable phenotypic variability in our cohort. To our knowledge, CENPI mutations have not been previously reported in men, making our study the first to describe its mutation in affected siblings. Functional studies have shown that CENPI mutations disrupt centromere integrity, leading to meiotic inversion and, consequently, reproductive barriers and infertility [68–73]. In our cohort, one patient with a CENPI mutation presented with meiotic arrest and a complete absence of spermatozoa, while another exhibited hypospermatogenesis with viable sperm retrieval. These findings underscore the considerable phenotypic diversity associated with CENPI mutations, complicating genotype–phenotype correlations. Notably, the patients’ responses to testicular sperm extraction (TESE) were highly variable, with one patient achieving successful retrieval while the other did not.

Phenotypic variability in NOA can be challenging to interpret due to the diverse anomalies observed not only within the same individual but also among individuals with the same mutation. This variability arises from multiple contributing factors. One key factor is histological intra-testicular heterogeneity, which is well-documented in the literature [74]. For instance, a large-scale study demonstrated that, in cases of Sertoli cell-only syndrome (SCOS), the phenotypic classification of right and left testicular biopsies was consistent in most cases. However, this alignment was less frequent in instances of meiotic arrest [75]. This suggests that testicular histopathology can display notable differences even when the underlying genetic mutation is identical in both testes.

Moreover, the cellular microenvironment within distinct regions of the testis plays a critical role in regulating spermatogenesis [76]. While certain regions exhibit robust spermatogenic activity, others demonstrate either a complete absence of spermatogenesis or its arrest. This regional variability highlights that the local testicular microenvironment significantly influences spermatogenic outcomes, even in the presence of identical genetic mutations [26]. Consequently, this reinforces the importance of considering both testicular tissue and the local microenvironment in diagnostic and therapeutic strategies. Furthermore, well-established concepts in human genetics, such as variable expressivity and penetrance, suggest that individuals with the same mutation may exhibit markedly different testicular phenotypes and spermatogenic outcomes [77, 78]. This variability is not limited to genetic factors alone, as digenic inheritance may contribute to the observed discrepancies in phenotypic expression. Additionally, genetic compensation, epigenetic modifications, or post-translational mechanisms could mitigate the phenotypic consequences of specific gene mutations [79]. These compensatory mechanisms may obscure the full extent of the genetic defect, adding complexity to the genotype–phenotype relationship. Environmental factors, such as endocrine disruptors or other exogenous influences, may also interact with the genetic background, further modulating spermatogenic outcomes [80].

This introduces another layer of complexity in understanding the genotype–phenotype correlation in NOA. Clinically, the primary goal of genetic diagnosis in NOA patients is to optimize TESE procedures by providing targeted guidance for more effective interventions. Understanding the multifactorial origins of phenotypic variability can improve the accuracy and predictability of these procedures, ultimately enhancing the success of sperm retrieval in NOA patients.

In cases involving gene mutations, where the prognosis for TESE (testicular sperm extraction) success remains uncertain, these mutations should be classified as uncertain, and TESE should not be excluded as a treatment option. Such findings emphasize the need for a cautious interpretation of genetic testing in the context of reproductive outcomes, as the relationship between genotype and phenotype remains complex and multifaceted.

Our research, consistent with many other studies, found that all individuals with mutations in meiotic genes (*TERB1*, *MEIOB*, and *SPO11*) had negative TESE outcomes, strongly suggesting an association between meiotic gene defects and poor TESE success. This association provides stronger grounds for assigning a negative prognosis to individuals with defects in meiotic genes, given the consistency of these findings across the cohort. While predicting TESE success based solely on genetic testing is inherently challenging due to the multifactorial nature of spermatogenesis, it is evident that genes related to meiosis offer more reliable prognostic information. These genes contribute to a more accurate and informed diagnosis, allowing clinicians to make better decisions regarding sperm retrieval strategies.

In the continuation of our study, we have planned functional assay experiments for the genes *HIRIP3* and *FTHL17*, which were identified outside of the panel we developed. In the initial phase, RNA sequencing (RNA-seq) is being conducted.

## Conclusion

Our study highlights the significant role of genetic analysis in understanding the complex etiology of NOA. By incorporating WES and comprehensive phenotypic evaluations, we identified multiple gene mutations associated with distinct testicular histologies, offering valuable insights into the genetic underpinnings of this condition. The study also underscores the importance of refining diagnostic strategies, with a focus on genes related to the testicular phenotype, which may help guide more accurate predictions for testicular sperm extraction success.

## Supplementary Information

Below is the link to the electronic supplementary material.

Supplementary file1 Table 1. Literature-Based Overview of NOA Candidate Genes (XLSX 367 KB)

Supplementary file2 Prediction Databases (DOCX 29 KB)

Supplementary file3 Information on the Cohort Study with Genetic Analysis (PDF 1235 KB)

## Data Availability

The datasets generated and/or analyzed during the current study are available within the manuscript and its supplementary materials. Additional information can be obtained from the corresponding authors upon reasonable request.
