# Supplementary material for: Genetic insights into non-obstructive azoospermia: Implications for diagnosis and TESE outcomes
Source: J Assist Reprod Genet. 2025 Feb 11;42(4):1223–37. doi: 10.1007/s10815-025-03409-5 (PMC12055743; doi:10.1007/s10815-025-03409-5)
Supplement: Supplementary file 2 — Supplementary file2 Prediction Databases (DOCX 29 KB) [file 10815_2025_3409_MOESM2_ESM.docx]

**Prediction Databases:**

American College of Medical Genetics (ACMG) pathogenicity (1)

REVEL (2)

MetaLR (3)

SIFT (4)
Polyphen2 (HumDiv) (5)
MutationTaster (6)
MetaSVM (3)
M-CAP (7)
MVP (8)
Provean (9)
BayesDel addAF (10)
BayesDel noAF (10)
FATHMM-XF (11)

DANN 0.9995 (12)

MutationAssessor (13)
CADD (PHRED-scaled) (14)

GME (15)

ClinPred (16)

**References**

1. Richards S, Aziz N, Bale S, Bick D, Das S, Gastier-Foster J, et al. Standards and guidelines for the interpretation of sequence variants: a joint consensus recommendation of the American College of Medical Genetics and Genomics and the Association for Molecular Pathology. Genet Med. 2015;17(5):405-24.

2. Ioannidis NM, Rothstein JH, Pejaver V, Middha S, McDonnell SK, Baheti S, et al. REVEL: An Ensemble Method for Predicting the Pathogenicity of Rare Missense Variants. Am J Hum Genet. 2016;99(4):877-85.

3. Dong C, Wei P, Jian X, Gibbs R, Boerwinkle E, Wang K, Liu X. Comparison and integration of deleteriousness prediction methods for nonsynonymous SNVs in whole exome sequencing studies. Hum Mol Genet. 2015;24(8):2125-37.

4. Ng PC, Henikoff S. SIFT: Predicting amino acid changes that affect protein function. Nucleic Acids Res. 2003;31(13):3812-4.

5. Adzhubei IA, Schmidt S, Peshkin L, Ramensky VE, Gerasimova A, Bork P, et al. A method and server for predicting damaging missense mutations. Nat Methods. 2010;7(4):248-9.

6. Schwarz JM, Rödelsperger C, Schuelke M, Seelow D. MutationTaster evaluates disease-causing potential of sequence alterations. Nat Methods. 2010;7(8):575-6.

7. Jagadeesh KA, Wenger AM, Berger MJ, Guturu H, Stenson PD, Cooper DN, et al. M-CAP eliminates a majority of variants of uncertain significance in clinical exomes at high sensitivity. Nat Genet. 2016;48(12):1581-6.

8. Qi H, Zhang H, Zhao Y, Chen C, Long JJ, Chung WK, et al. MVP predicts the pathogenicity of missense variants by deep learning. Nat Commun. 2021;12(1):510.

9. Choi Y, Sims GE, Murphy S, Miller JR, Chan AP. Predicting the functional effect of amino acid substitutions and indels. PLoS One. 2012;7(10):e46688.

10. Feng BJ. PERCH: A Unified Framework for Disease Gene Prioritization. Hum Mutat. 2017;38(3):243-51.

11. Shihab HA, Gough J, Cooper DN, Stenson PD, Barker GL, Edwards KJ, et al. Predicting the functional, molecular, and phenotypic consequences of amino acid substitutions using hidden Markov models. Hum Mutat. 2013;34(1):57-65.

12. Quang D, Chen Y, Xie X. DANN: a deep learning approach for annotating the pathogenicity of genetic variants. Bioinformatics. 2015;31(5):761-3.

13. Reva B, Antipin Y, Sander C. Predicting the functional impact of protein mutations: application to cancer genomics. Nucleic Acids Res. 2011;39(17):e118.

14. Kircher M, Witten DM, Jain P, O'Roak BJ, Cooper GM, Shendure J. A general framework for estimating the relative pathogenicity of human genetic variants. Nat Genet. 2014;46(3):310-5.

15. Scott, E.M., et al., Characterization of Greater Middle Eastern genetic variation for enhanced disease gene discovery. Nat Genet, 2016. 48(9): p. 1071-6

16. Alirezaie, N., et al., ClinPred: Prediction Tool to Identify Disease-Relevant Nonsynonymous Single-Nucleotide Variants. Am J Hum Genet, 2018. 103(4): p. 474-483.

**Web resources**

ClinVar, <https://www.ncbi.nlm.nih.gov/clinvar>/

GenBank, <https://www.ncbi.nlm.nih.gov/genbank>/

gnomAD Browser, <https://gnomad.broadinstitute.org/>

1000Genome, <https://www.internationalgenome.org/>

MGI database, <http://www.informatics.jax.org>/

OMIM, <https://www.omim.org>/

Varsome, <https://varsome.com>/

Ensembl, <https://www.ensembl.org/>

dbSNP, <https://www.ncbi.nlm.nih.gov/snp/>

[Entrez](https://www.ncbi.nlm.nih.gov/gene/2200), <https://www.ncbi.nlm.nih.gov/Web/Search/entrezfs.html>

HGNC, <https://www.genenames.org/>

SIFT 4G, <https://sift.bii.a-star.edu.sg/sift4g/>

VEST4, <https://jhu.technologypublisher.com/technology/24805>

PrimateAI, <https://primateai3d.basespace.illumina.com/>

LIST-S2, <https://list-s2.msl.ubc.ca/?session=F97BC494C8B3ACFD0B12703890D63B4D>

ExAC, <https://avillach-lab.hms.harvard.edu/exome-aggregation-consortium-exac>

UK10K, <https://www.uk10k.org>

STRING, https://string-db.org

MGI, https://www.informatics.jax.org

DISEASES , <https://diseases.jensenlab.org/Search>

DAVID bioinformatics, <https://davidbioinformatics.nih.gov/tools.jsp>

GTEx, <https://gtexportal.org/home/>

Franklin , <https://franklin.genoox.com/clinical-db/home>

Proteinatlas, https://www.proteinatlas.org/humanproteome/tissue/testis
