# Supplementary material for: Genetic insights into non-obstructive azoospermia: Implications for diagnosis and TESE outcomes
Source: J Assist Reprod Genet. 2025 Feb 11;42(4):1223–37. doi: 10.1007/s10815-025-03409-5 (PMC12055743; doi:10.1007/s10815-025-03409-5)
Supplement: Supplementary file 3 — Supplementary file3 Information on the Cohort Study with Genetic Analysis (PDF 1235 KB) [file 10815_2025_3409_MOESM3_ESM.pdf]

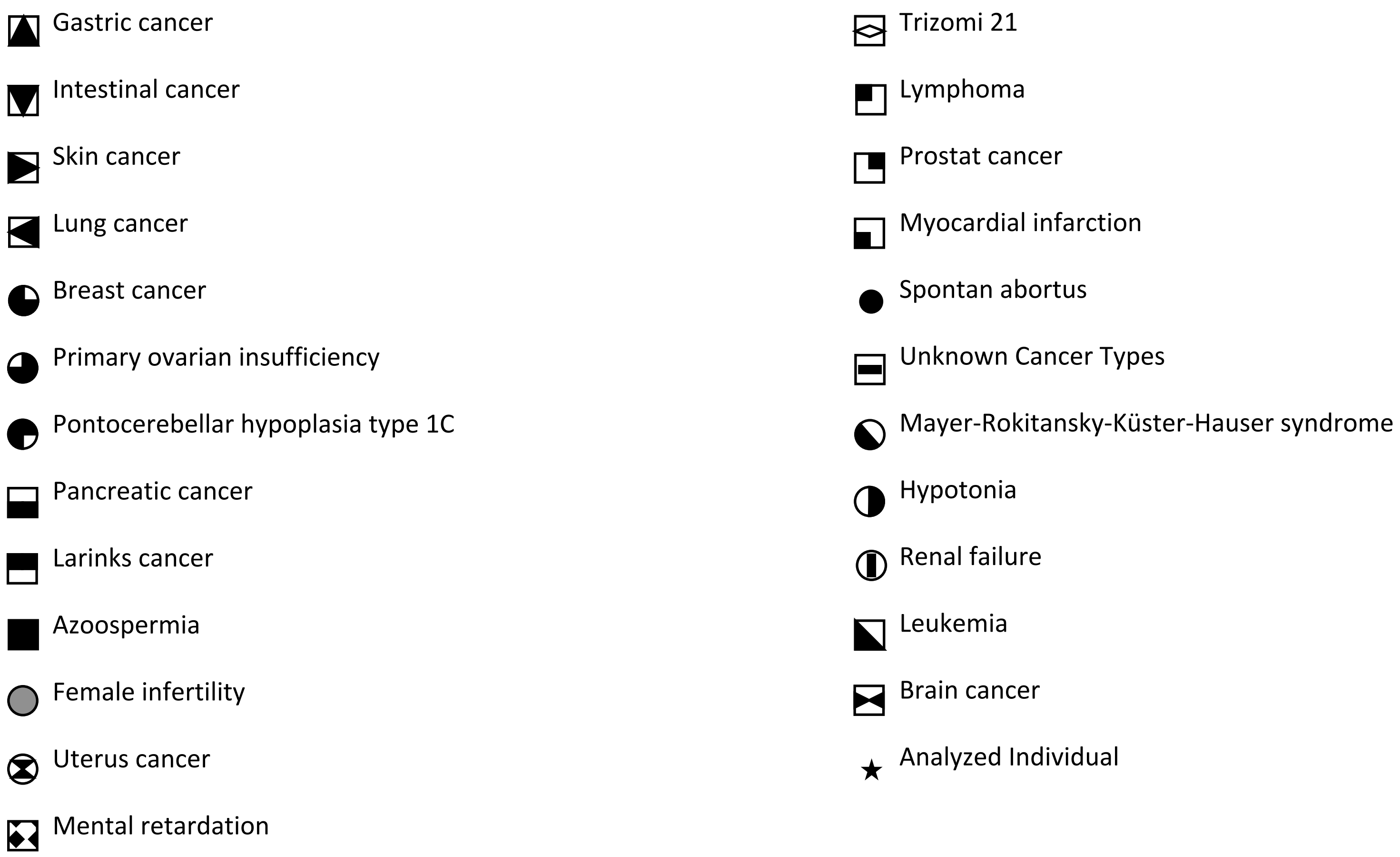

*Pedigree Symbols Used to Indicate Clinical Information

Information on Families with Identified Potential Candidate Variants

F 1

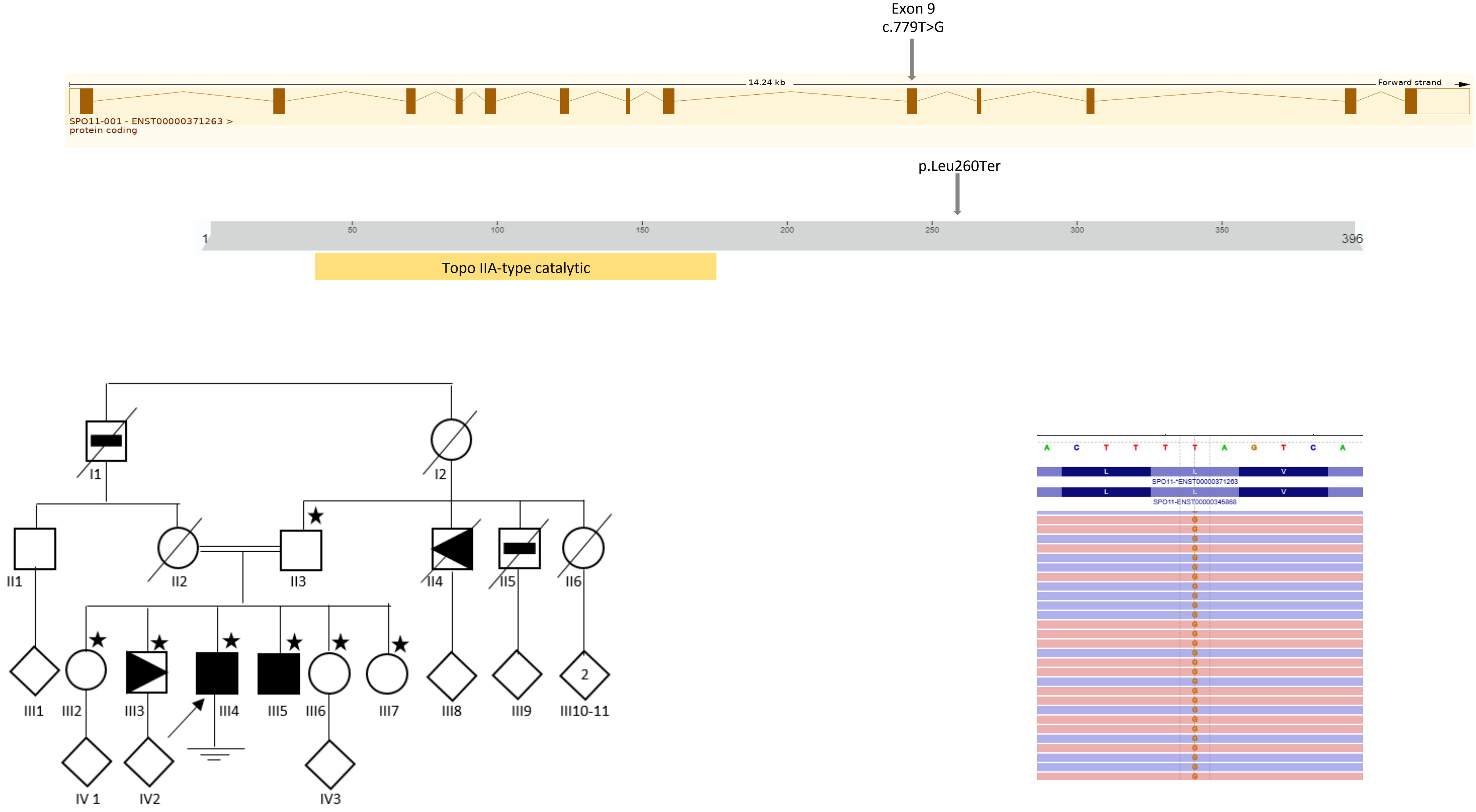

SPO11 INITIATOR OF MEIOTIC DOUBLE-STRANDED BREAKS; *SPO11*

HGVSc: ENST00000371263.3:c.779T>G

F 2

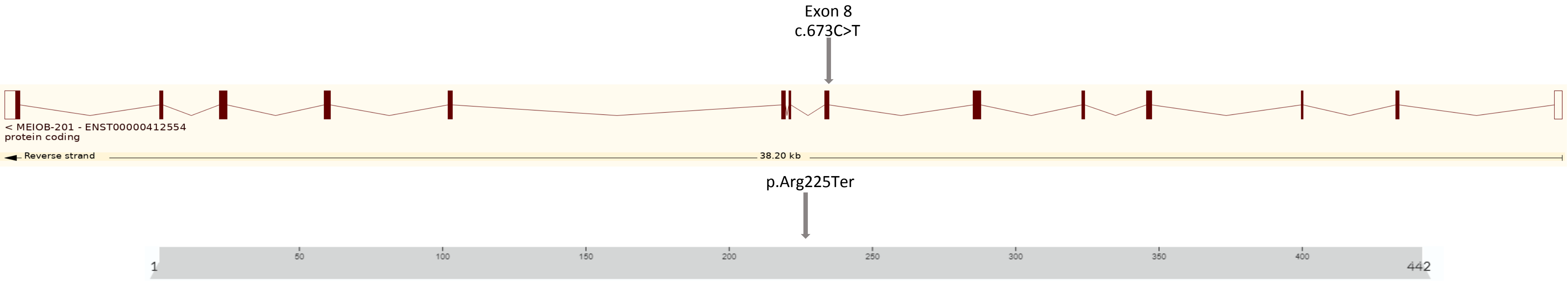

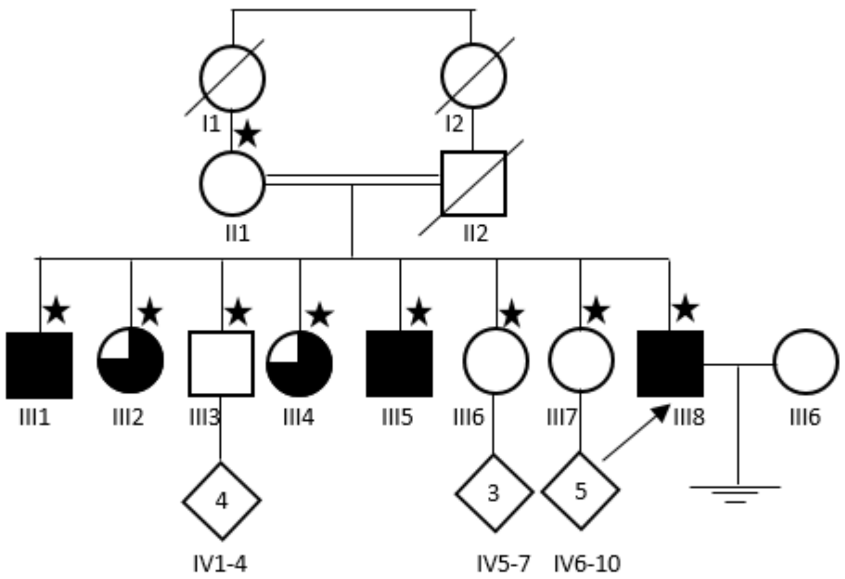

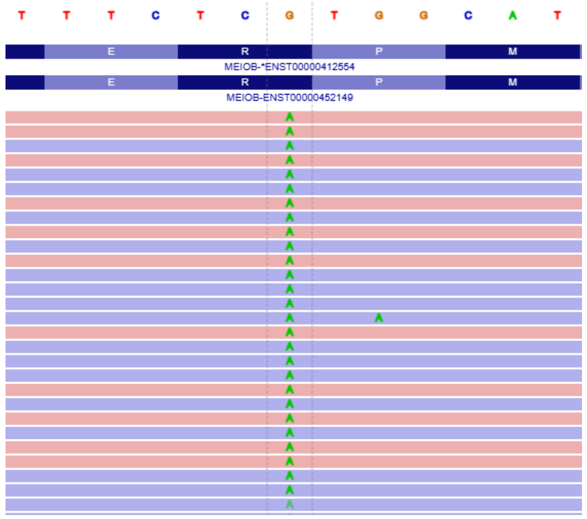

MEIOSIS-SPECIFIC PROTEIN WITH OB DOMAINS; *MEIOB*

HGVSc: ENST00000412554.2:c.673C>T

F 3

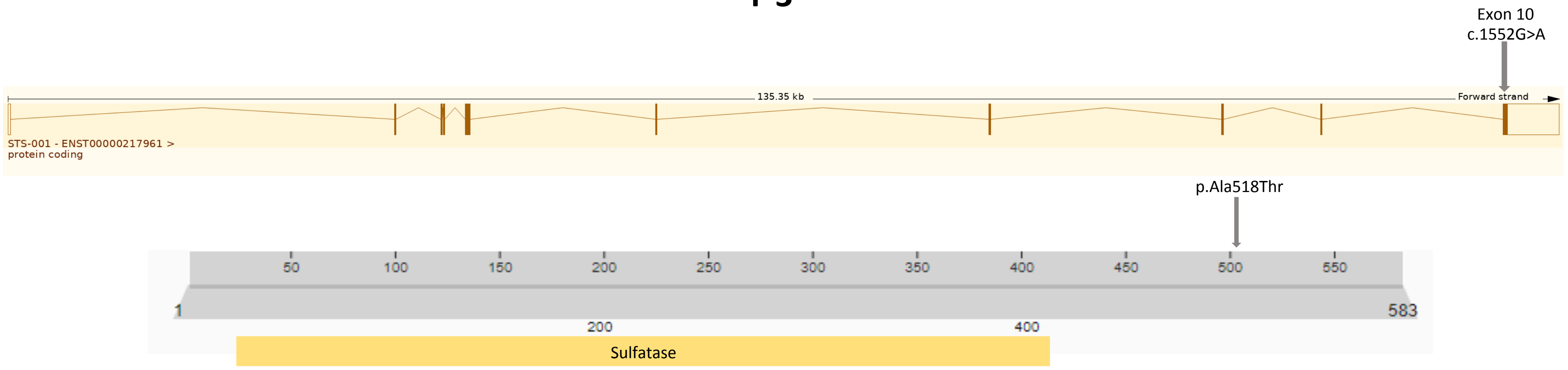

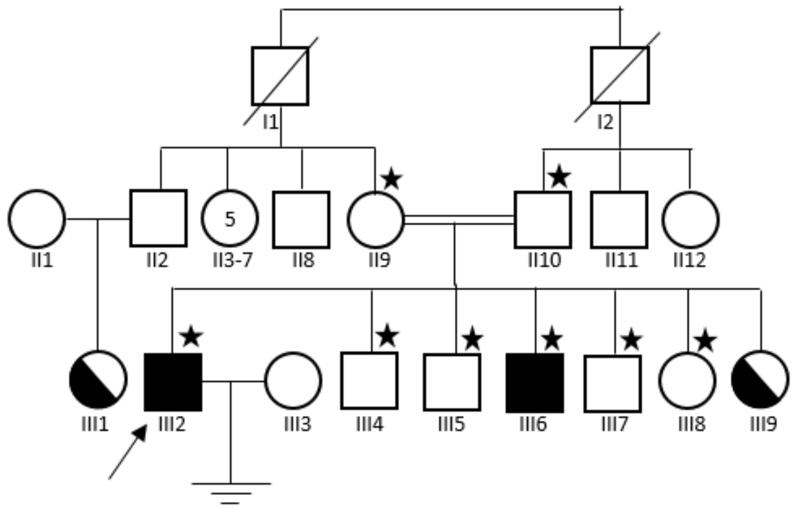

STEROID SULFATASE; *STS*

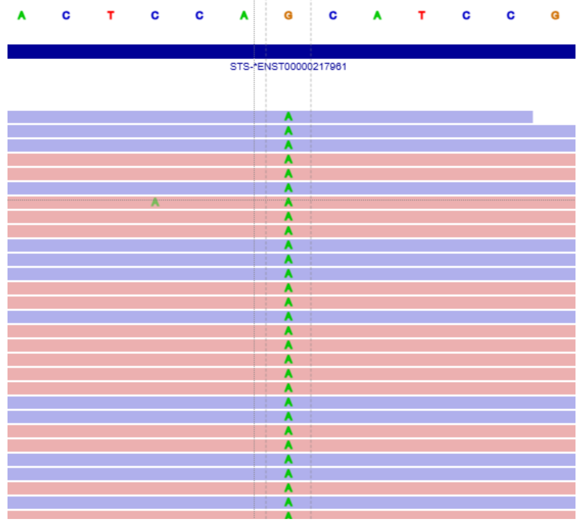

HGVSc: ENST00000217961.4:c.1552G>A

F 4

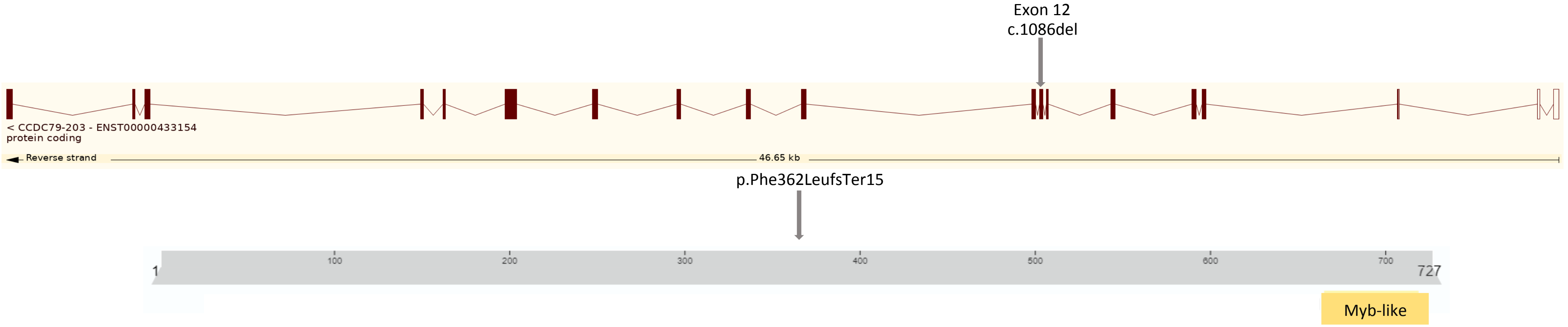

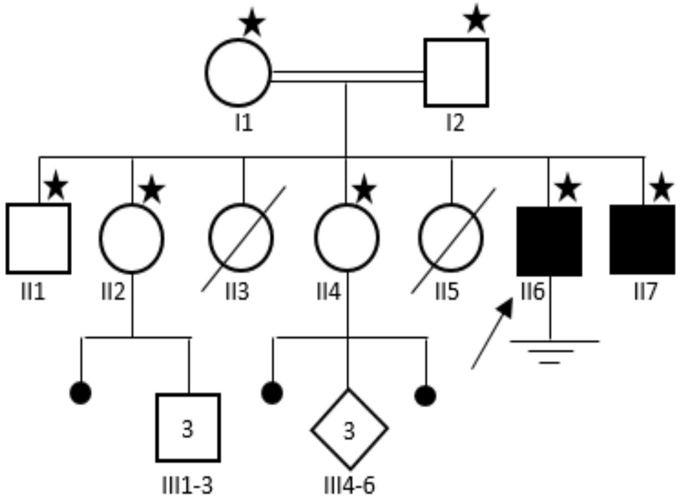

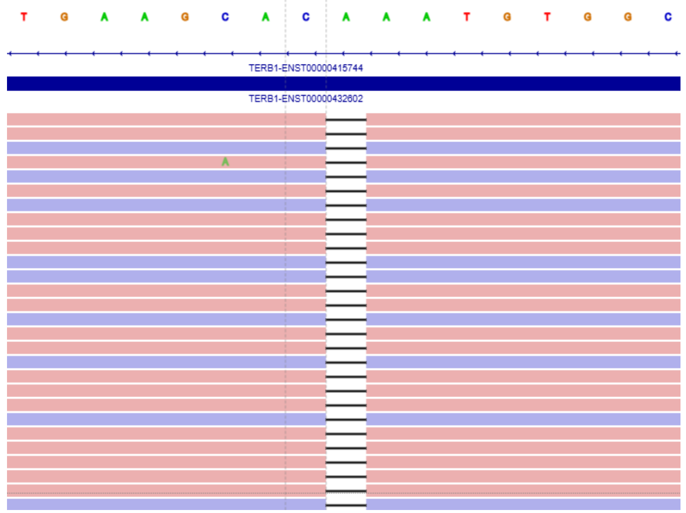

TELOMERE REPEAT-BINDING BOUQUET FORMATION PROTEIN 1; *TERB1*

HGVSc: ENST00000433154.1:c.1086del

F 5

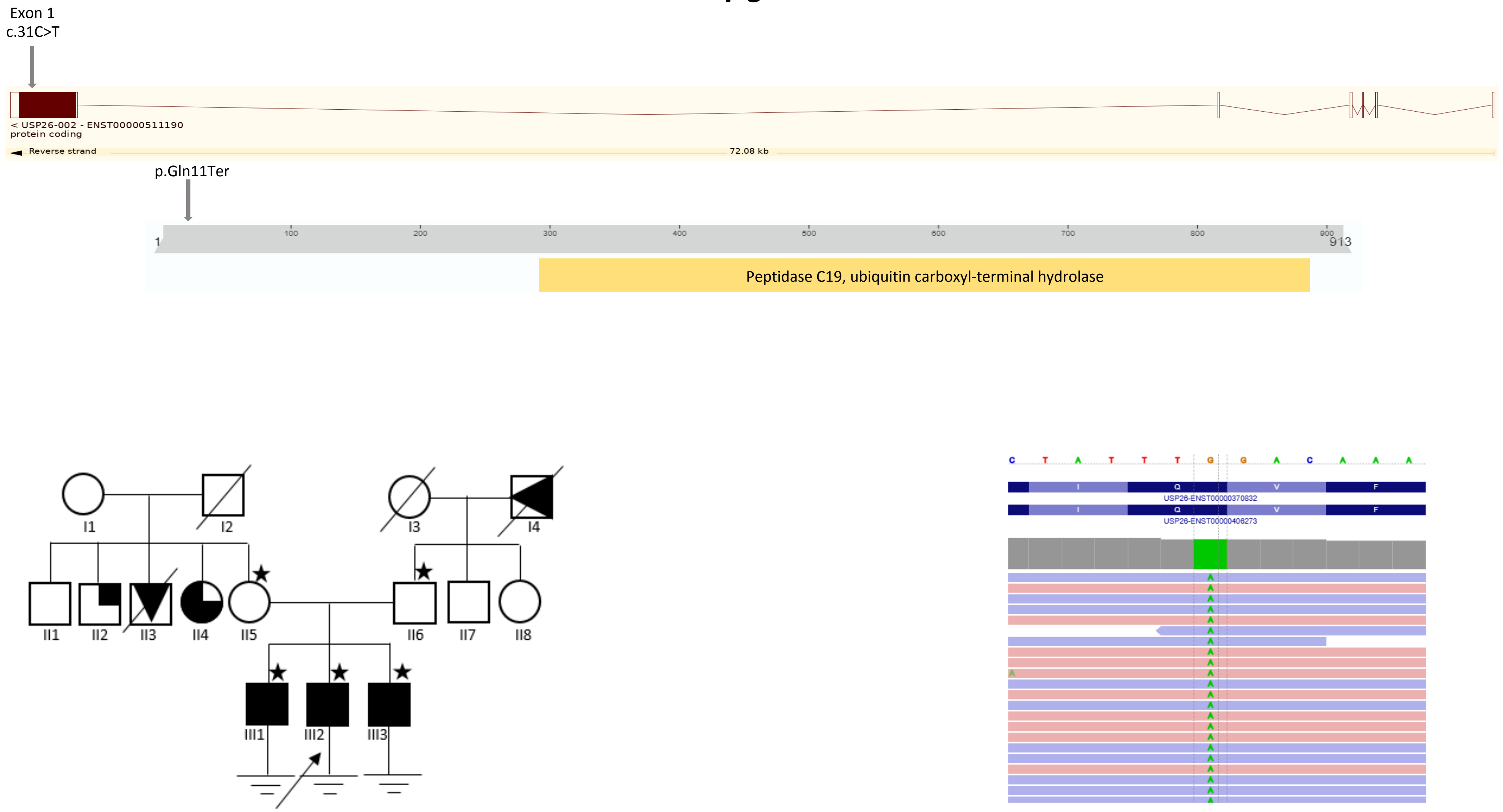

UBIQUITIN-SPECIFIC PROTEASE 26; *USP26*

HGVSc: ENST00000511190.1:c.31C>T

F 6

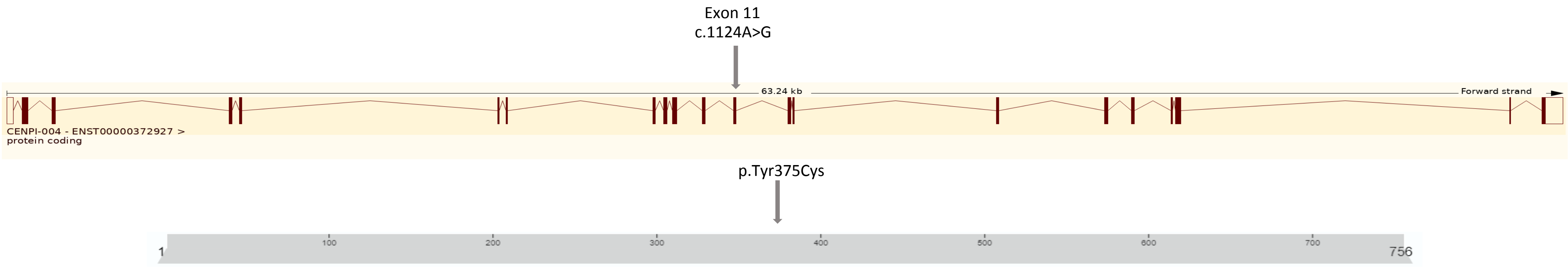

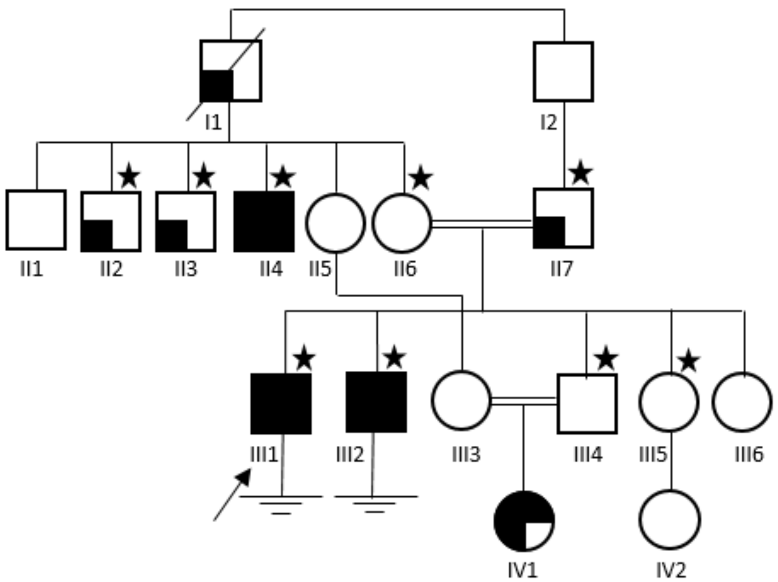

CENTROMERIC PROTEIN I; *CENPI*

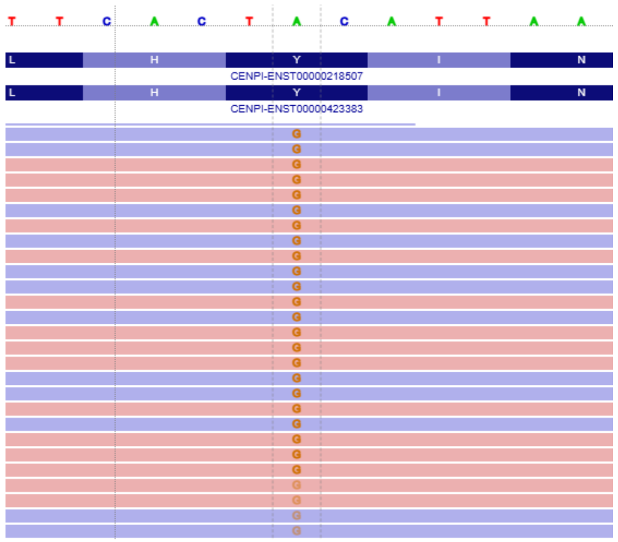

HGVSc: ENST00000372927.1:c.1124A>G

F 7

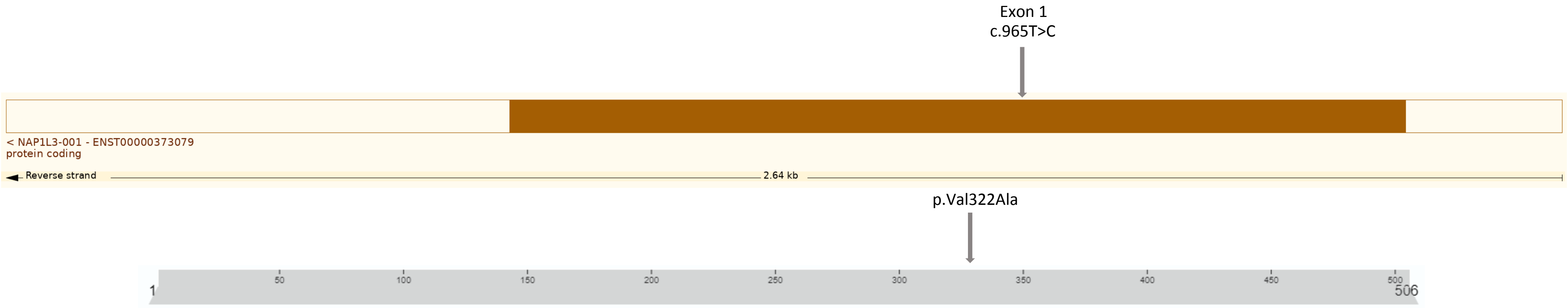

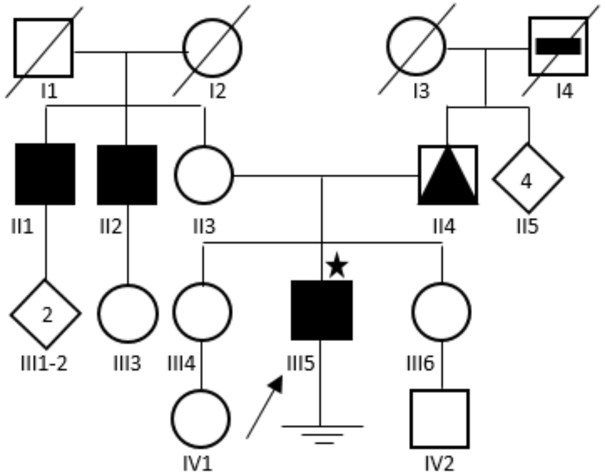

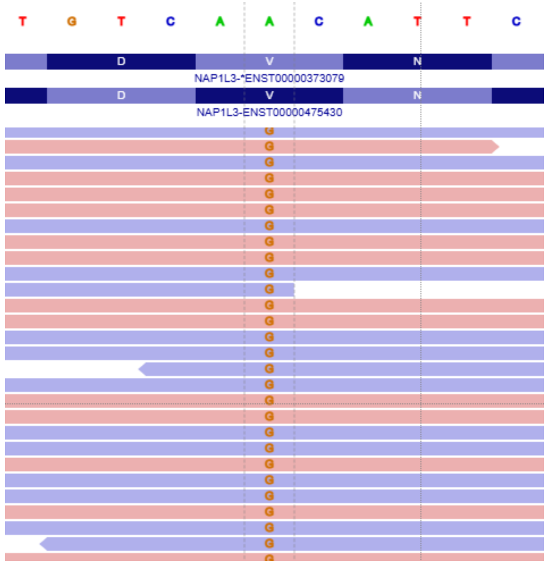

NUCLEOSOME ASSEMBLY PROTEIN 1-LIKE 3; *NAP1L3*

HGVSc: ENST00000373079.3:c.965T>C

F 8

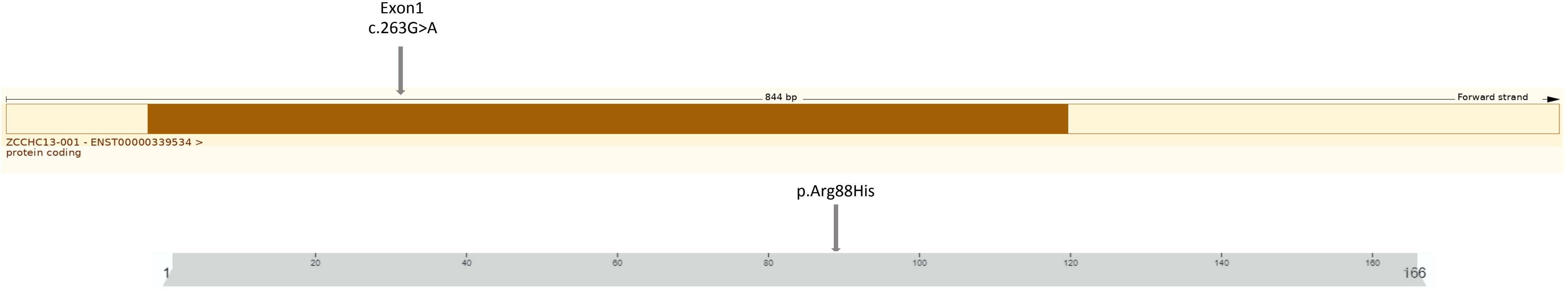

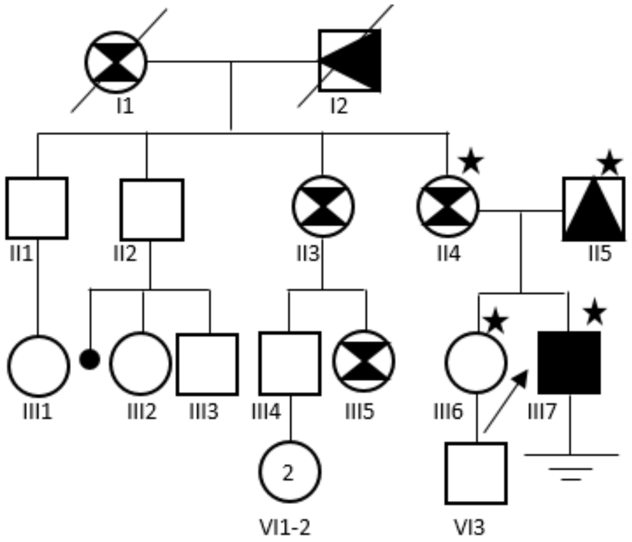

ZINC FINGER, CCHC DOMAIN CONTAINING 13; *ZCCHC13*

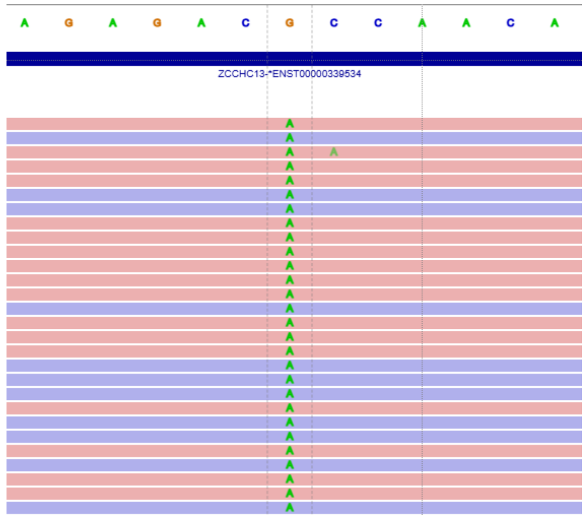

HGVSc: ENST00000339534.2:c.263G>A

F 9

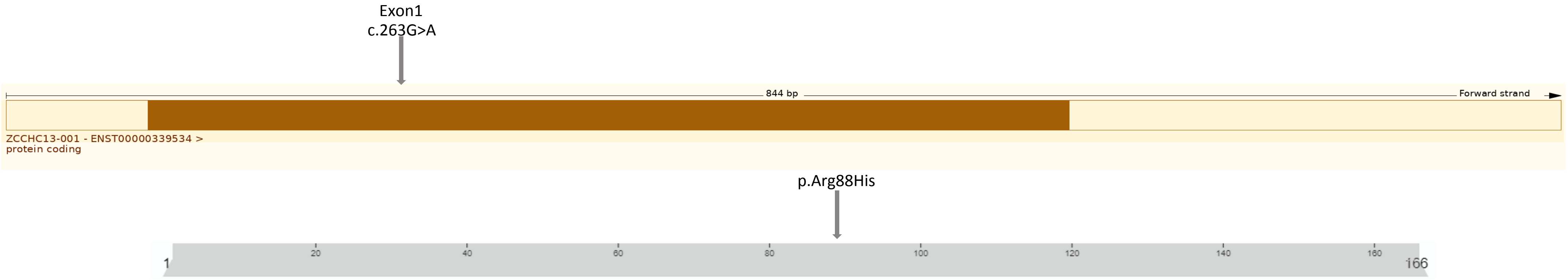

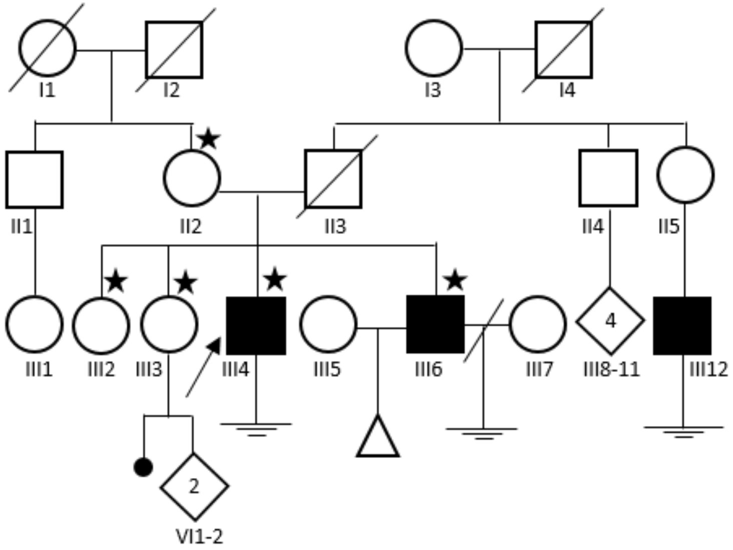

RETINOBLASTOMA-BINDING PROTEIN 7; *RBBP7*

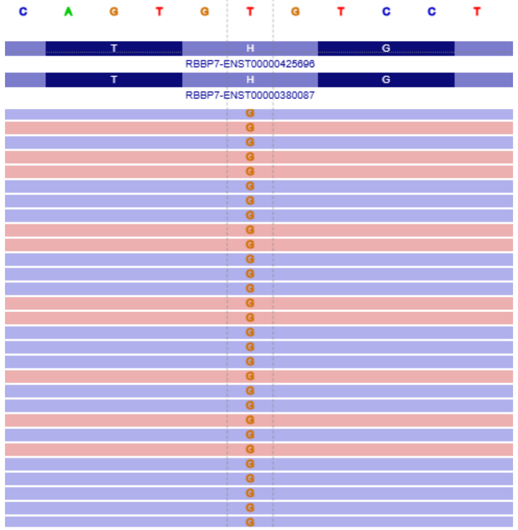

HGVSc: ENST00000380084.4: c.1247A>C

F 10

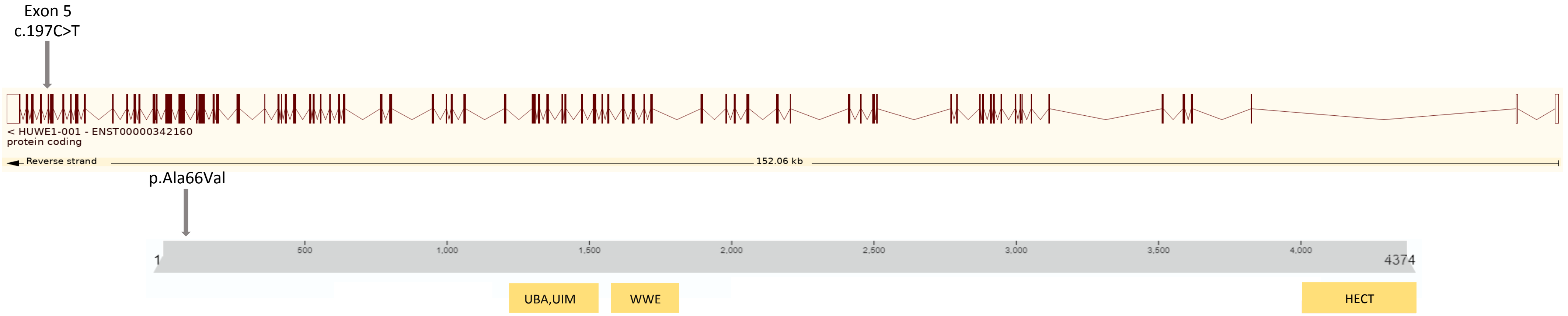

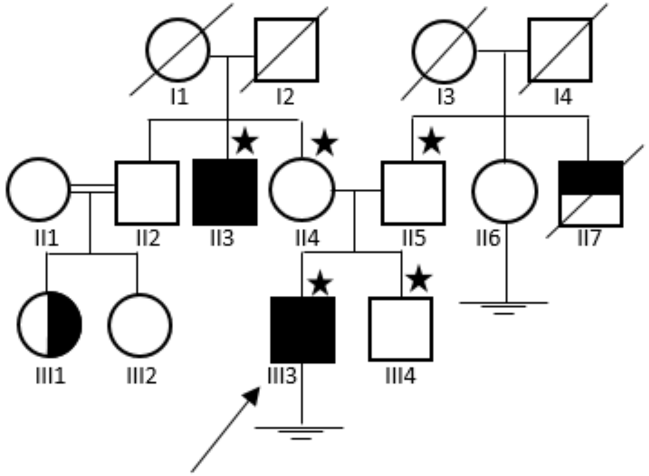

HECT, UBA, AND WWE DOMAINS-CONTAINING PROTEIN 1; *HUWE1*

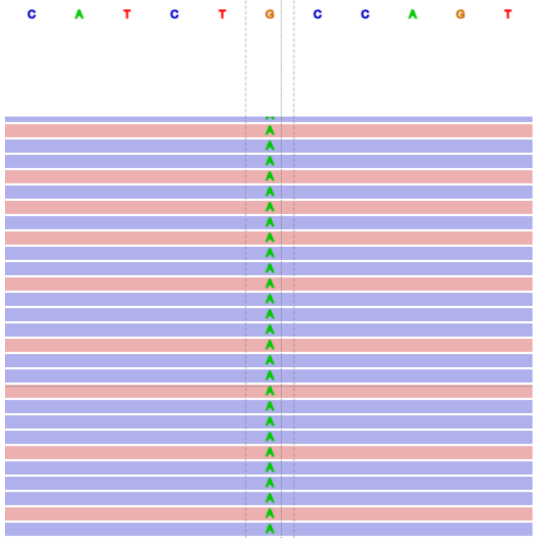

HGVSc: ENST00000342160.3: c.197C>T

F 11

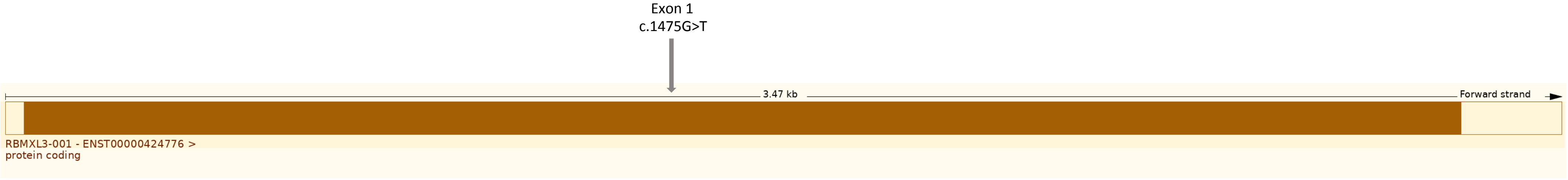

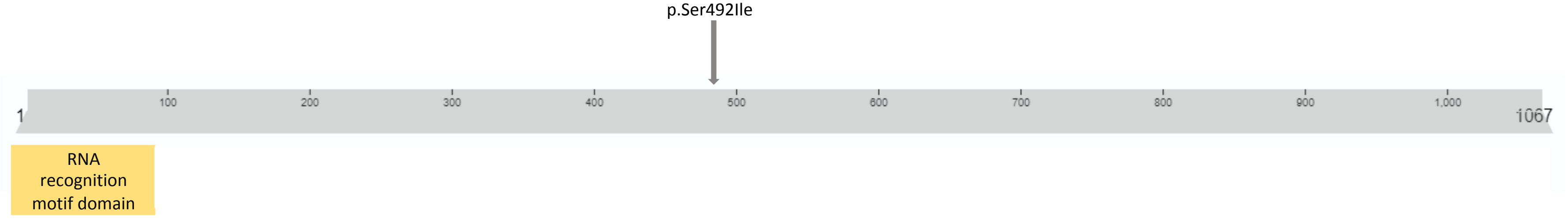

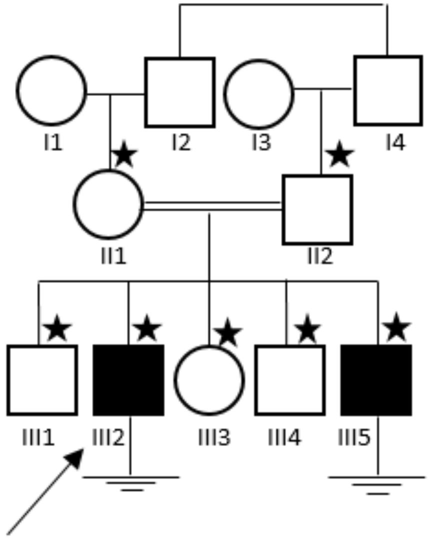

RNA-BINDING MOTIF PROTEIN, X-LINKED-LIKE-3; *RBMXL3*

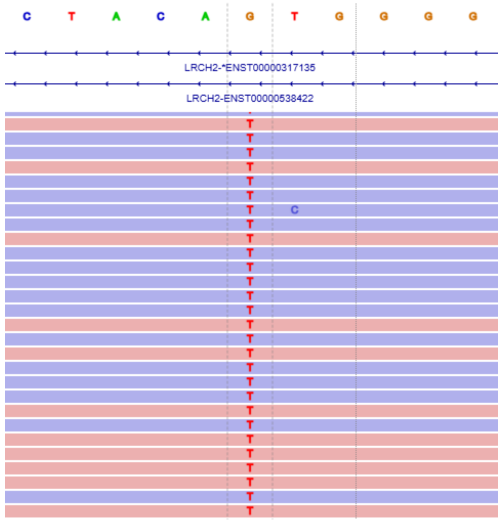

HGVSc: ENST00000424776.3: c.1475G>T

F 12

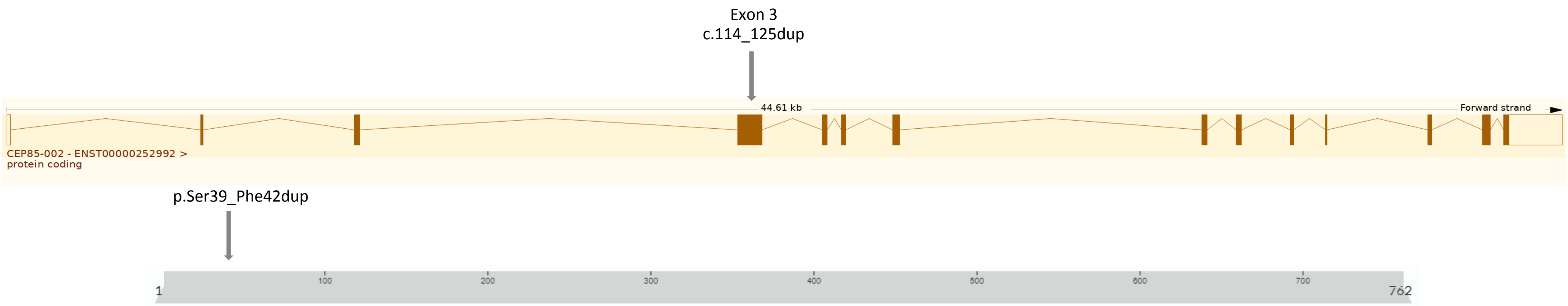

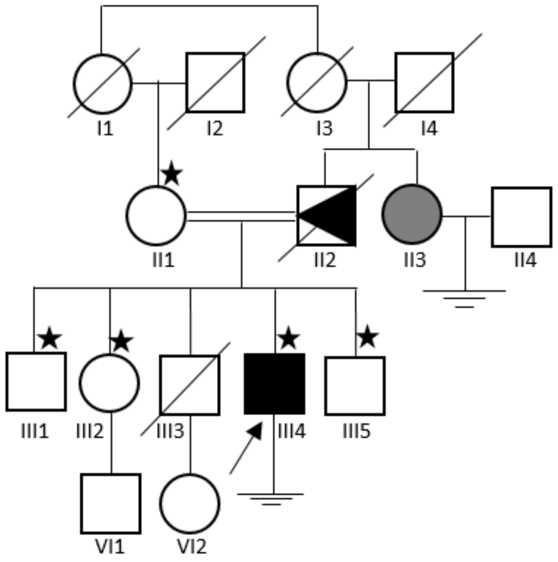

CENTROSOMAL PROTEIN, 85-KD; *CEP85*

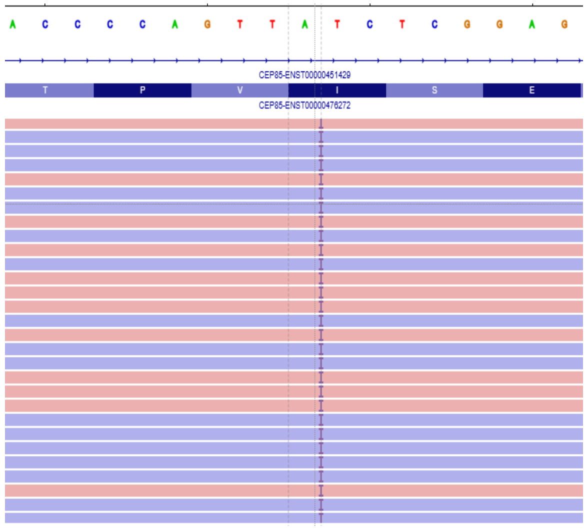

HGVSc: ENST00000252992.4:c.114_125dup

F 13

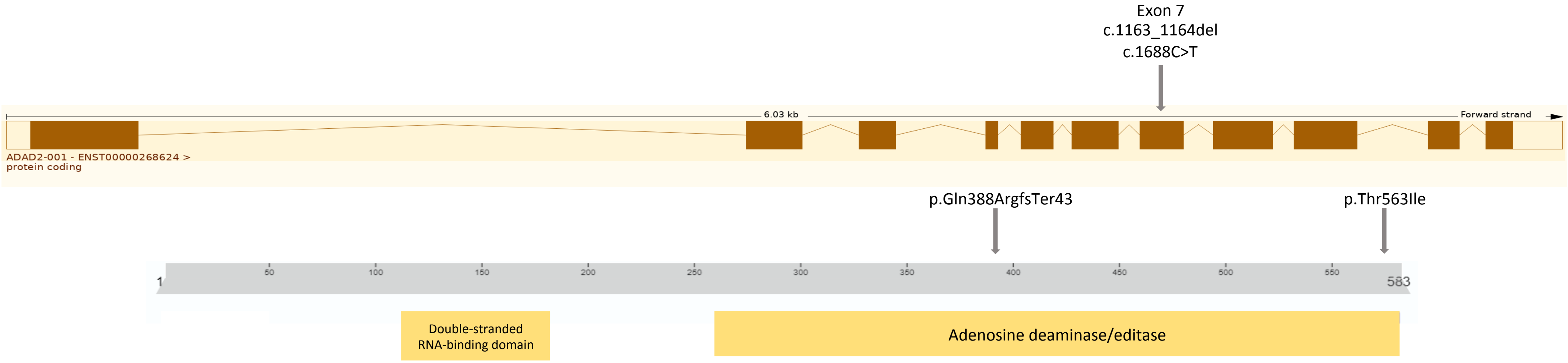

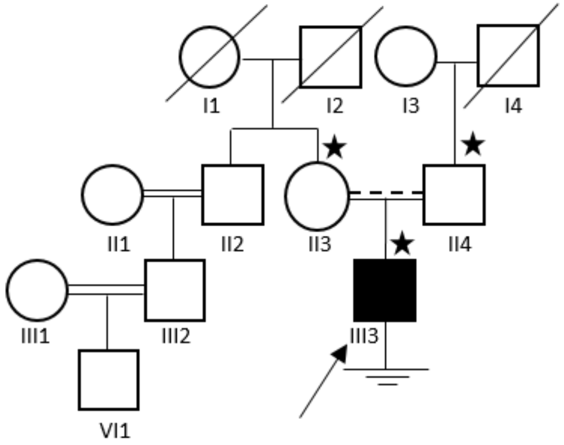

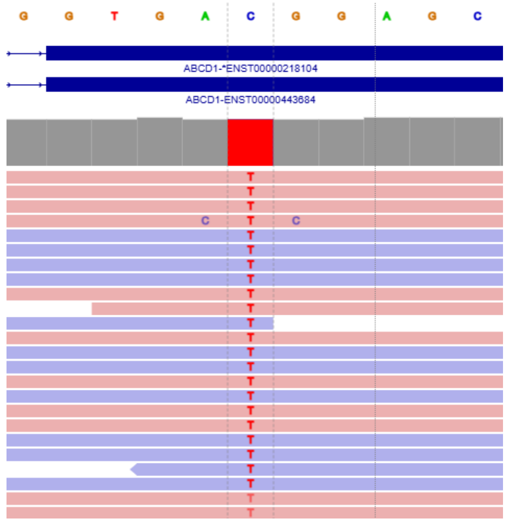

ATP-BINDING CASSETTE, SUBFAMILY D, MEMBER 1; *ABCD1*

HGVSc: ENST00000218104.3:c.1229C>T

F 14

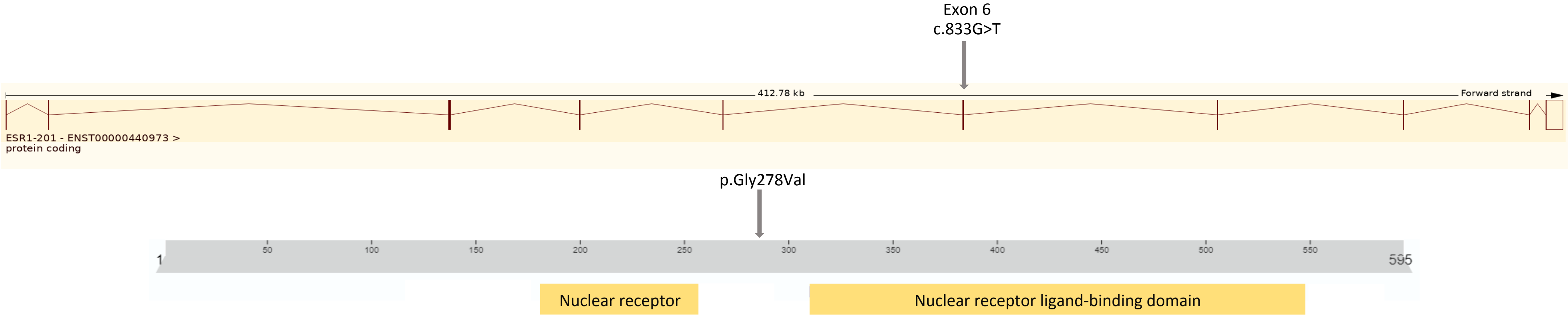

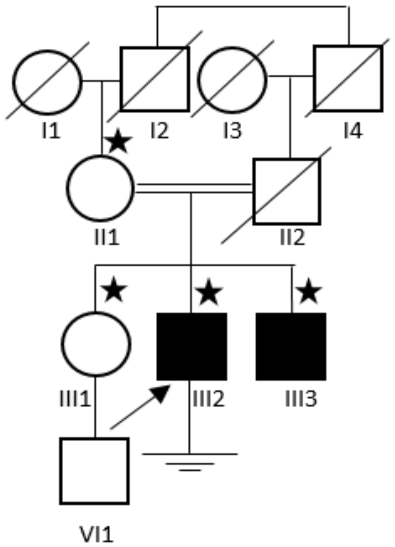

ESTROGEN RESISTANCE, ESTROGEN RECEPTOR 1; *ESR1*

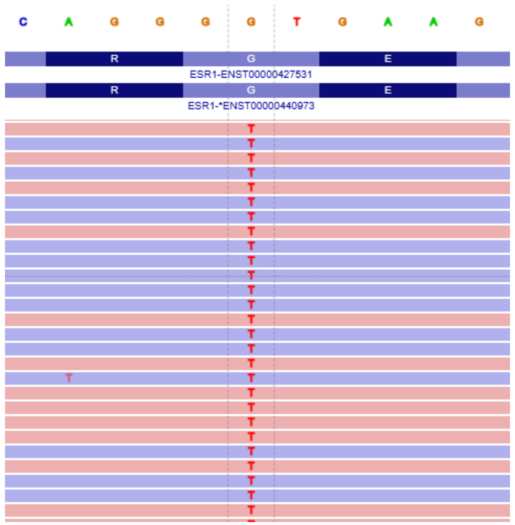

HGVSc: ENST00000440973.1:c.833G>T

Genes as Potential Candidates for Functional Studies

F 15

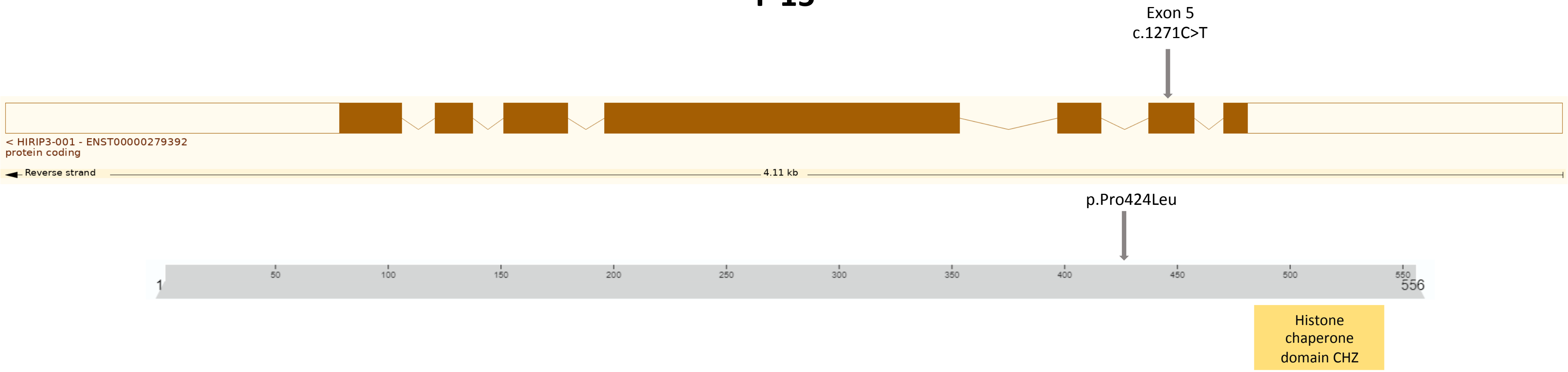

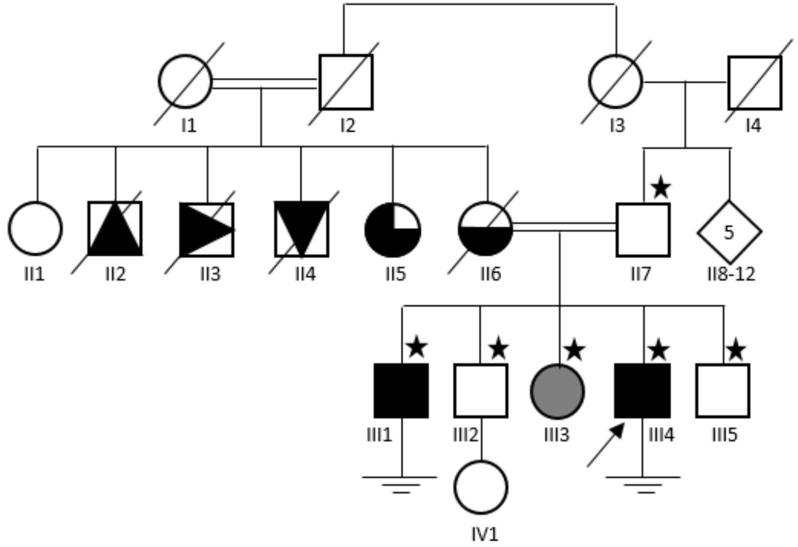

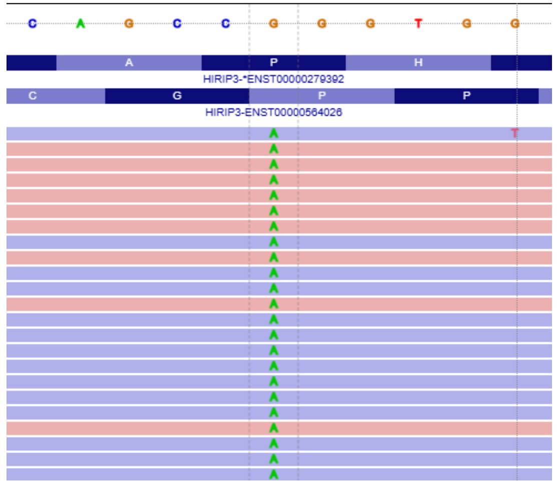

HIRA-INTERACTING PROTEIN 3; *HIRIP3*

HGVSc: ENST00000279392.3:c.1271C>T

F 16

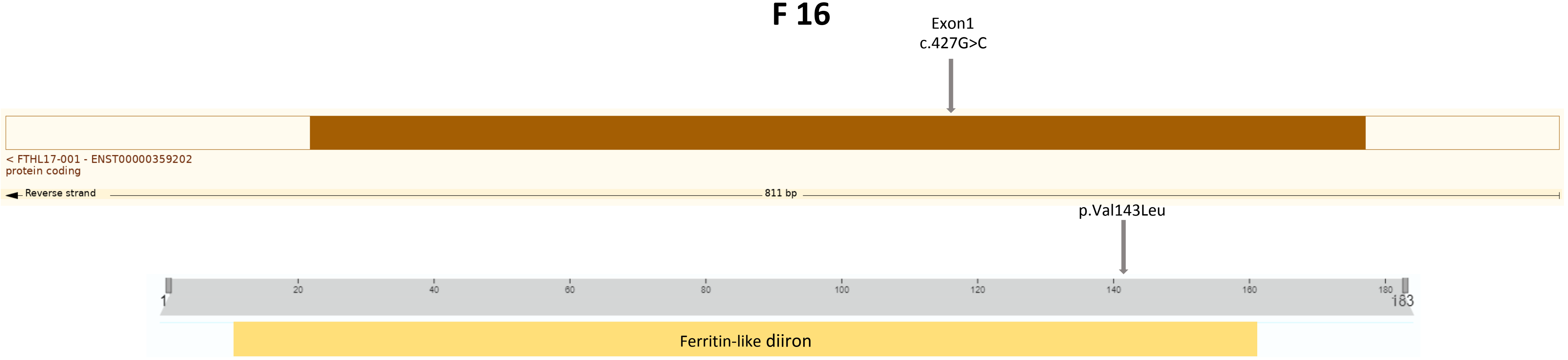

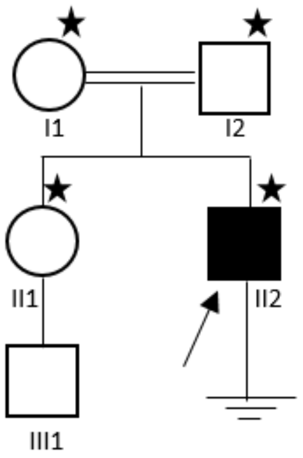

FERRITIN HEAVY POLYPEPTIDE-LIKE 17, *FTHL17*

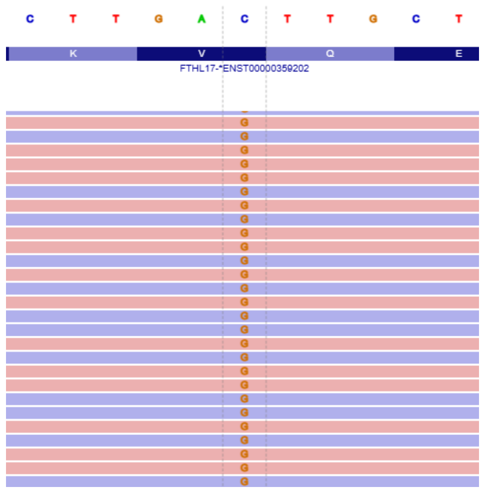

HGVSc: ENST00000359202.3:c.427G>C

Information on Families with No Identified Potential Candidate Variants

F 17

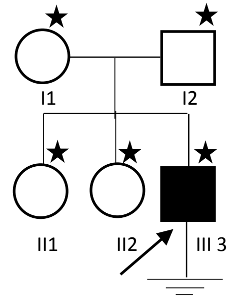

F 18

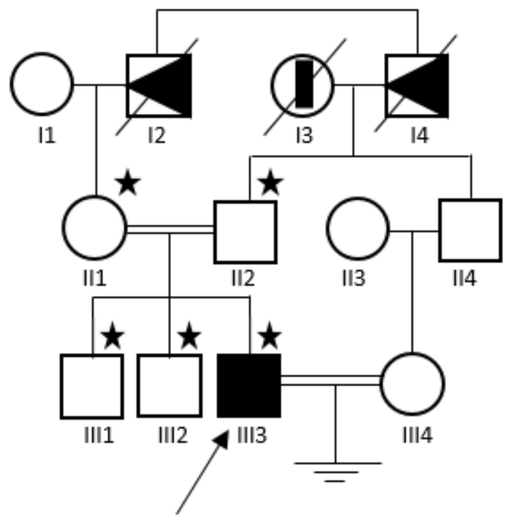

F 19

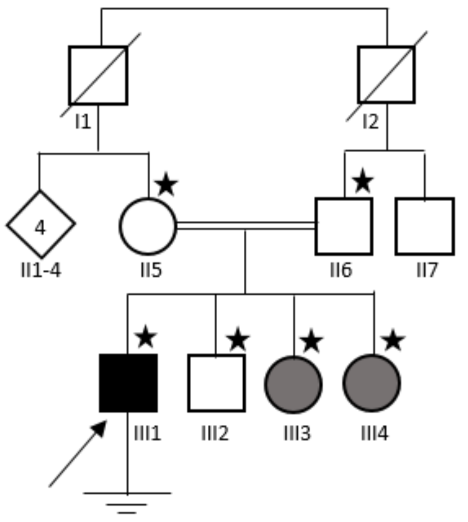

F 20

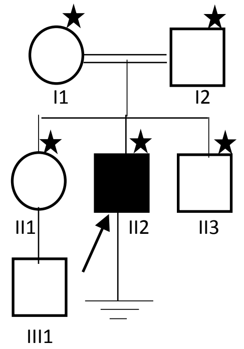

F 21

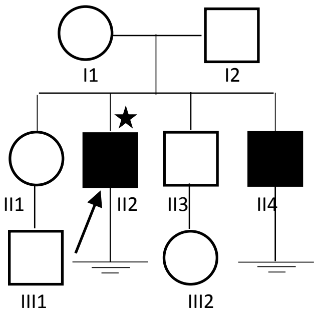

F 22

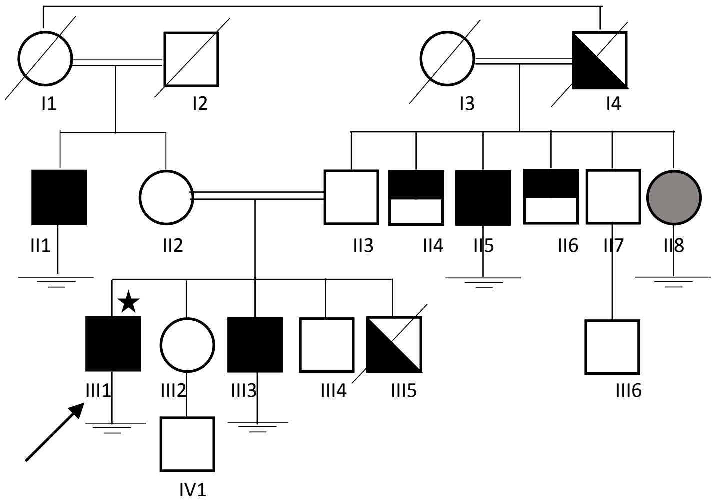

F 23

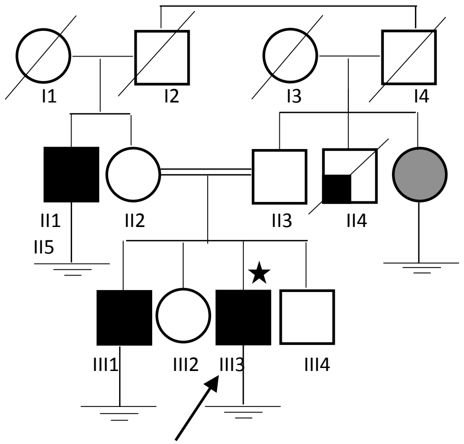

F 24

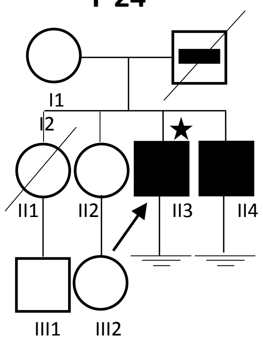

F 25

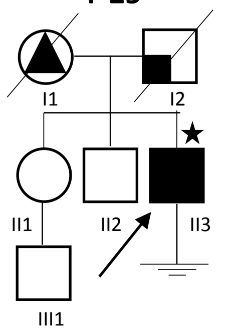

F 26

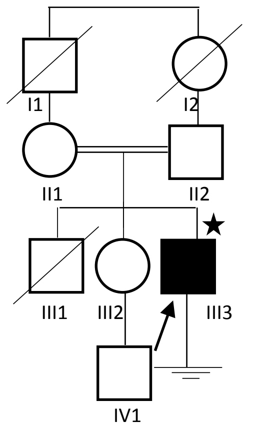

F 27

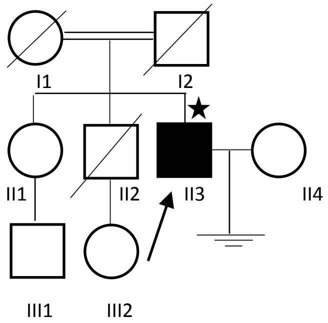

F 28

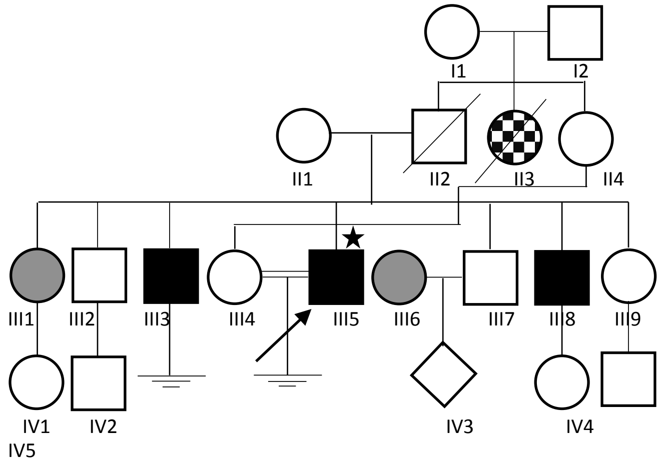

F 29

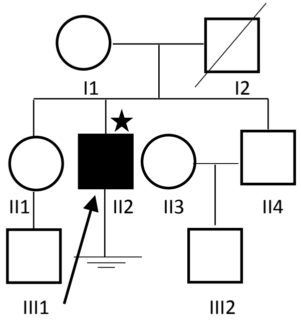

F 30

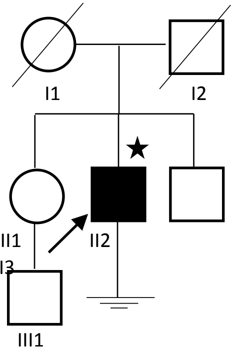

F 31

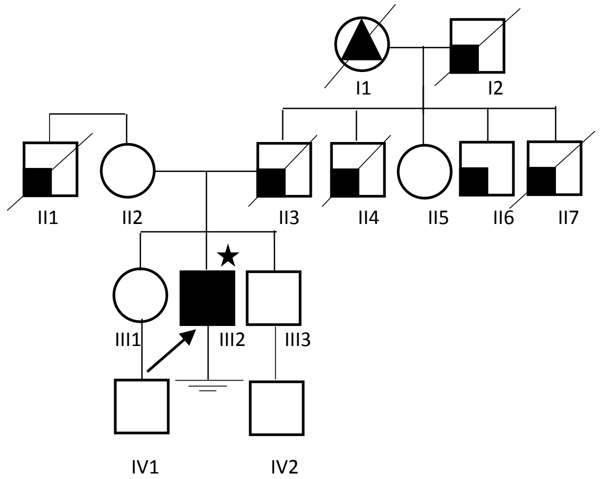

F 32

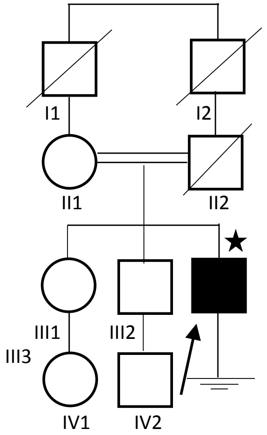

F 33

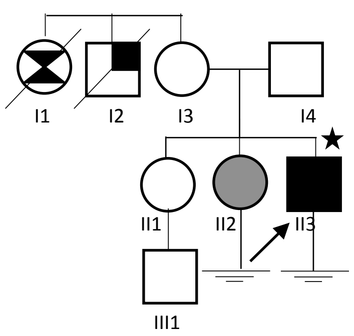

F 34

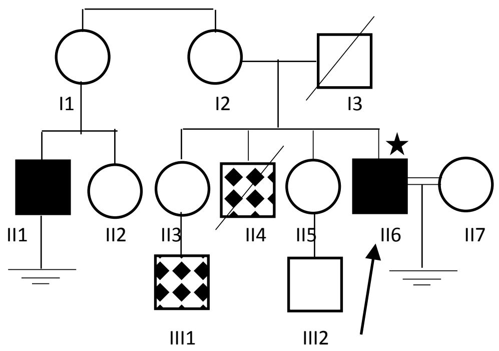

F 35

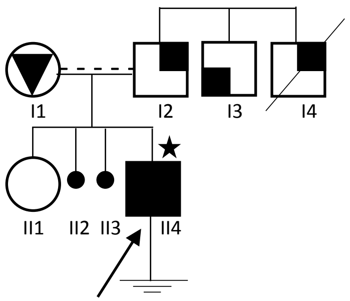

F 36

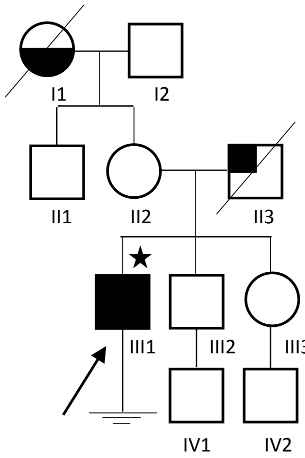

F 37

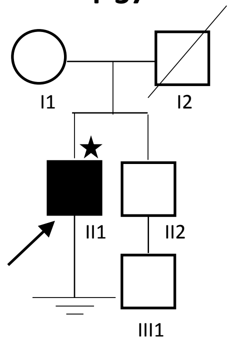

F 38

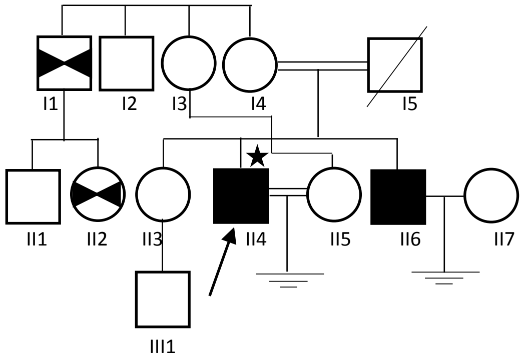

F 39

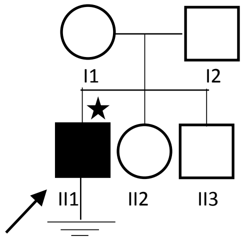

F 40

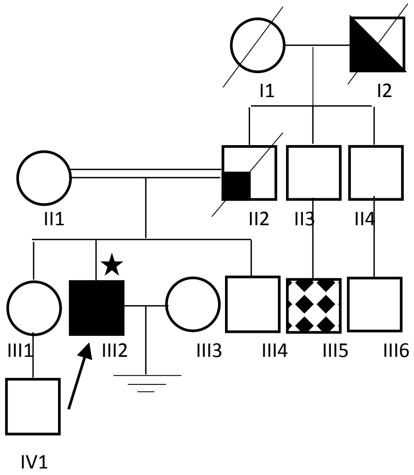

F 41

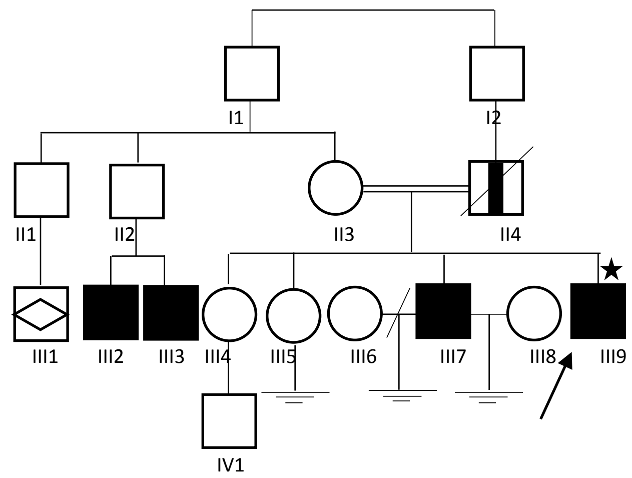

F 42

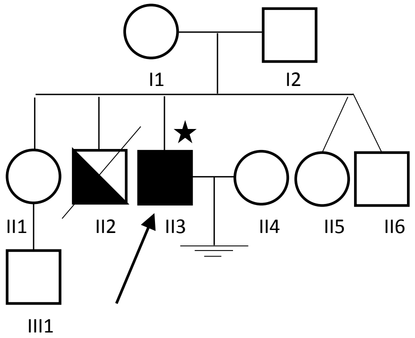

**For individuals F43, F44, F45, F46, F47, F48, and F49, family history information is unavailable
